# Is substrate choice an overlooked variable in ecotoxicology experiments?

**DOI:** 10.1007/s10661-023-10935-1

**Published:** 2023-01-30

**Authors:** Georgia M. Sinclair, Michela Di Giannantonio, Oliver A. H. Jones, Sara M. Long

**Affiliations:** 1 Australian Centre for Research on Separation Science (ACROSS), School of Science, RMIT University, PO Box 71, Bundoora West Campus, Bundoora, VIC 3083 Australia; 2 National Research Council (CNR-IAS), Institute for the study of Anthropic Impacts and Sustainability in Marine Environment, Genoa, Italy; 3 Aquatic Environmental Stress (AQUEST) Research Group School of Science, RMIT University, Bundoora, VIC 3083 Australia

**Keywords:** Metabolic profiling, Toxicology, Method development, Copper, Amphipods

## Abstract

It is crucial to understand the effects caused by experimental parameters such as temperature, light, and food type on lab and field-based ecotoxicology experiments, as these variables, and combinations thereof, can affect results. The type of substrate used in exposure experiments, however, is generally assumed to have no effect. This may not always be correct. The metabolic changes in the freshwater crustacean, *Austrochiltonia subtenuis* exposed to copper, using three common substrates, gauze; toilet paper; and cellulose were investigated. Substrate alone did not affect survival, but each substrate elicited a different metabolic response and adult and juvenile amphipods had different substrate preferences. Several classes of metabolites were shown to change in response to different substrates and toxicant. These included disaccharides, monosaccharides, fatty acids, and tricarboxylic acid cycle intermediates. The results illustrate that metabolomic responses can differ in response to experimental factors that were previously thought not to be significant. In fact, our data indicate that substrate should be viewed as an experimental factor as important to control for as more well-known confounders such as temperature or food, thus challenging the current paradigm. Assuming substrate type has no effect on the experiment could potentially lead to errors in contaminant toxicity assessments. We propose that ideal good practise would be that all experimental factors should be evaluated for their potential influence on metabolomic profiles prior to contaminant response experiments being undertaken.

**Supplementary Information:**

The online version contains supplementary material available at 10.1007/s10661-023-10935-1.

## Introduction

Ecotoxicity studies must be consistent, reliable, and reproducible, to provide the scientific community and regulators with useful data on contaminant toxicity, and facilitate development of ‘safe’ guideline values (Ruden et al., 2016). To this end, many testing guidelines are in place, such as CRED (Criteria for Reporting and Evaluating Ecotoxicity Data Project) or OECD guidelines (Moermond et al., 2016), that aim to produce a framework to follow for peer-reviewed papers for test set-up, and data reporting (Savić-Zdravković et al., 2020). It is crucial to follow such guidelines when designing an experiment, but not to follow them blindly. It is equally important to understand that there may be potential confounders in a particular experiment which may not be accounted for in the guidelines. For example, it is known that changes in parameters such as food, temperature or light regime may result in changes in the sensitivity of an organism to a contaminant (Spurgeon et al., 2010). Similarly, the use of field-collected sediment as a substrate for aquatic organisms in toxicology tests may be environmentally relevant but can also increase the variability of the experiment. This is because it is almost impossible to obtain sediment that is uniform in concentrations of nutrients and free from trace contaminants that may interact with the specific stressor(s) under study. For this reason, alternatives to environmental sediments, such as the use of cellulose or gauzes, are often used in ecotoxicology experiments. These substrates are generally assumed to be inert and so their potential effect on a test organism’s sensitivity to contaminants are not often assessed. We suggest this may be a mistake.

Currently, there is no preferred option for substrates used in exposure experiments. For example, cotton gauzes are commonly used for amphipods (Everitt, MacPherson, Brinkmann, Wiseman, & Pyle, 2020; Jeppe et al., 2017a, b; Moore & Farrar, 1996; Schlechtriem et al., 2019; Vu et al., 2017). An alternative is ethanol rinsed supermarket toilet paper, which has been typically used in exposures with *Chironomus*, (non-biting midges) (Jeppe et al., 2014; Long et al., 2015; Mehler et al., 2017; Townsend et al., 2012). A third possible substrate is powdered cellulose (Gagliardi et al., 2015; Marinković et al., 2011). All these substrates are thought to be inert to organism metabolism but what if they aren’t? How can this be tested?

Metabolomics is the study of small molecule metabolites such as amino acids, sugars, and organic acids, and how they change in response to external stimuli in biological systems (Jones et al., 2013). It can be used to highlight metabolic responses to a wide range of factors, including environmental contaminants (Viant, 2009; Zhang et al., 2021). Environmental metabolomics has been applied to a wide range of organisms, such as invertebrates (Sinclair et al., 2019a, b), crustaceans (Vandenbrouck et al., 2010), fish (Tsentalovich, Zelentsova, Yanshole, Yanshole, & Odud, 2020) and reptiles (Beale et al., 2021), following exposure to manmade and naturally occurring compounds (Matich et al., 2019). The techniques can potentially lead to the identification of biochemical biomarkers of contaminant exposure. For example, Long et al., (2015) identified changes in methionine, cystathionine and lanthionine in *Chironomus tepperi* larvae, as potential biomarkers of zinc exposure, resulting from impacts on the transsulfuration pathway from zinc. Jeppe and et al., (2017a, b) later suggested specific changes in the transsulfuration pathway in could be used as biomarkers of exposure of aquatic invertebrates to heavy metals following a field-based microcosm exposure experiment with copper. Other workers have shown metabolomics to be a useful method to test the effects of experimental parameters (Hillyer, Beale, & Shima, 2021).

To assess if substrate choice might affect the metabolic response elicited by a contaminant, the biochemical baseline responses of a common test organism, the amphipod *Austrochiltonia subtenuis*, following two experiments was investigated. The first experiment investigated the impact of different substrates (gauze; toilet paper; and cellulose)*.* The second experiment assessed the effect of exposure to the heavy metal copper (Cu) and the effect of the three different substrates from the first experiment. Survival rates were recorded following exposure. Metabolites levels were then assessed using Gas Chromatography-Mass Spectrometry (GC–MS). A number of metabolite groups, including disaccharides, monosaccharides, fatty acid and conjugates, and Tricarboxylic Acids (TCA) cycle intermediates, were identified and shown to change in response to both substrate type and copper exposure.

The aim of this research was to highlight the importance of accounting for substrate type in ecotoxicology experiments and demonstrating the utility of metabolomics for assessing subtle effects that may be caused by experimental factors previously thought to be constant. Additionally, the use of biochemical approaches, such as metabolomics, to validate experimental design, particularly in ecotoxicology, is highlighted.

## Methods

### Chemicals and equipment

All chemicals and equipment were analytical grade, purchased from Sigma-Aldrich, (Bayswater, Victoria, Australia) and used as received unless otherwise indicated.

### Organism

The test organism was the amphipod *Austrochiltonia subtenuis.* Amphipods are a well-used and well characterised test organism in ecotoxicology*.* Individuals were originally collected (over 5 years ago) from a wild population in Deep Creek, Bulla Rd, Bulla, Victoria, Australia (S 37° 37.919157' E 144° 47.995837') at varying life-stages. They have since been cultured in-house at RMIT University (Melbourne, Victoria, Australia). The amphipods were maintained in aquaria using a standard artificial media (SAM) modified from Borgmann (1996). The SAM consisted of reverse osmosis (RO) water (18.2 mΩ), obtained in house, with 0.23 mM NaHCO_3_, 0.061 mM CaCl_2_, 0.032 mM MgSO_4_, 0.47 mM NaCl, 0.0087 mM KCl, 0.17 mM MgCl_2_ and 0.0009 mM NaBr. Cultures were maintained at 21 ± 1 °C under a 16:8 h light: dark photoperiod. The culture was fed with powdered fish food (Tetramin®, Tetra Werke, Melle, Germany) and yeast–cerophyll–trout chow (YCT) made-up inhouse every second day.

### Substrates

All the artificial test substrates are well established in ecotoxicology experiments and used to remove the variability of using environmental sediments. Cellulose was purchased from Sigma-Aldrich (St. Louis, Missouri, United States). Toilet paper was purchased from a local supermarket. It was rinsed thoroughly with 100% ethanol and left to dry under a fume hood to ensure it was as sterile as possible prior to use. Gauze was cut from pre-sterilized bandage (Livingstone, Mascot, New South Wales, Australia) obtained from a local chemist. NB. While it is possible that amphipods potentially ingested the substrates this scenario is thought to be unlikely as they were routinely fed their preferred food source throughout exposure so would have no need to ingest sediment directly.

### Substrate—only exposure

Five replicate beakers (600 mL) were used for each treatment. Gauze was cut into 2 × 2-cm squares, and two squares were added to each of the five replicates and topped up with 400 mL of SAM. A one ply sheet of toilet paper was roughly torn into quarters for each of the five replicates and the beakers topped up with SAM. Approximately 1.5 (± 0.05) g of powdered cellulose was weighed into each beaker, then topped up with SAM. A further five beakers with 400 mL of SAM without a substrate, i.e., water only, were used as a negative control for the experiment. All beakers were placed in a light (16-8 h cycle) and temperature-controlled room (20 $\pm$ 1 °C) overnight with constant aeration to allow the substrates to settle. On day one of the test, gravid amphipods (*n* = 200) were collected from culture tanks and 10 gravid, female amphipods were randomly added to each beaker.

During the exposure, amphipods were fed every second day with 1 mL powdered fish food solution (90 mg of ground Tetramin/50 ml reversed osmosis water) and 1 mL yeast–cerophyll–trout chow (YCT). The water was changed every week with fresh SAM. The quality of the water was checked at the very beginning of the exposure and at the end of each week before a water change (dissolved oxygen; conductivity; pH; temperature and ammonia) (supplementary material, Table S1).

On Day 14, post-gravid adult amphipods were removed and counted, placed in a pre-cooled microcentrifuge tube, and then quenched on dry ice and stored at − 80 °C until further analysis. Juveniles that were released by the female adults during the 14 days, were counted (total number for each replicate and survival are presented in the supplementary material, Table S2) and replicate samples were further separated to reduce space competition in beakers (for example, cellulose replicates were further separated by 50/50 into cellulose 1A and 1B (Total 97, 1A = 49 and 1B = 48 individuals)). All juvenile (< 14 days old) amphipods were at the same developmental stage during the experiments. Fresh substrates were added to each treatment and exposure continued for a further 28 days. Once again, amphipods were fed every second day and water was changed each week and the quality checked.

On day 28, the experiment was ended. The surviving juveniles were counted and then combined from each beaker into one tube, placed in a microcentrifuge tube and placed on dry ice for instant quenching of metabolites, and stored at − 80 °C prior to metabolomic analysis.

### Copper substrate exposure

Forty, 14-day old amphipods (male and female) were collected from the amphipod culture at RMIT University, placed in a microcentrifuge tube and quenched on dry ice at the commencement of the experiment. These samples were used as the Initial Baseline Control (IBC). Fifteen beakers (600 mL) were set up with 400 mL of SAM spiked with 5% of the LC_50_ for copper sulfate (CuSO_4_) (5% of LC_50_ 0.0605 µg/L), based on Australian and New Zealand marine and freshwater water quality guidelines for *Hyalella azteca* (a model species of freshwater amphipod) as these were the closest guidelines in relation to the organism used in this study (both species being from the order Amphipoda) (ANZG, 2018).

Five replicates were used per treatment. Each beaker contained either gauze, toilet paper or cellulose and were run alongside matched controls (the same substrate without copper). Forty, 14-day old amphipods were randomly added to each beaker and placed in a temperature-controlled incubator (16:8 h light: dark, (20 $\pm$ 1 °C) with gentle aeration, i.e., consistent light bubbles that do not disturb the substrate or break the water surface in the beakers) for 14 days and fed every two days with 1 mL powdered fish food (Tetramin®,) solution (90 mg of ground Tetramin / 50 ml reversed osmosis water) and 1 mL yeast–cerophyll–trout chow (YCT). After 7 days, water quality was recorded (supplementary material, Table S3) and 50% of the water was changed. Cellulose, toilet paper and gauze were collected from each replicate (with CuSO_4_, and without as controls for each substrate) at the end of the 14-day exposure and stored in -20 °C freezer prior to copper analysis.

At the end of the exposure, surviving amphipods were counted, pooled from each replicate to account for variance in sex, and placed in a microcentrifuge tube on dry ice for instant quenching of metabolites. Then stored in − 80 °C freezer until further analysis. The survival rate of each experiment was also recorded (supplementary material, table S2).

### Copper analyses of water and substrate

Water samples (50 mL) were collected at the beginning and at the end of the exposures. Samples were pooled from each copper and control treatment to determine the Copper (Cu) concentration (*n* = 6). The Cu concentration was measured using Inductively Coupled Plasma Mass Spectrometry (ICP-MS). For this, one milliliter of nitric acid was added to each test tube and heated to 80 °C on a heating block in large test tubes (50 mL) for > 2 h. Once water samples had cooled, they were transferred to 50-mL falcon tubes. The water samples were filtered through a 45 µm Syringe Filter and then made up to 50 mL using Milli Q water prior to analysis.

The concentration of copper was measured in the substrate from each replicate at the conclusion of the experiment by placing the substrates in glass test tubes and drying them in an oven at 60 °C for 48 h until completely dry. Dry substrates were then placed on a heating block and 200 µL of nitric acid was added to each sample and heated for 5 h. Once cool, 4 mL of Milli Q water was added, and the supernatant was collected into falcon tubes. The substrate samples were filtered through a 45-µm syringe filter and then made up to 5 mL using Milli Q water in preparation for the ICP-MS.

All samples were analyzed for copper using an Agilent 7700 × quadrupole ICP-MS (Agilent Technologies, Mulgrave, Australia), equipped with an Agilent ASX-520 autosampler following EPA Method 3051A (EPA and of Resource Conservation, 2007). The instrument was operated in He-mode. The integration time was 0.3 s per mass, 1 point per mass, 3 replicates, and 100 sweeps per replicate. The Agilent Environmental standard for ICP-MS (Agilent Technologies) was used for quantification (supplementary material, Table S4).

### Sample preparation and metabolite extraction

Metabolites were extracted from each sample by first adding 600 µL of methanol: chloroform (9:1 ratio). Samples were sonicated for 15 min in an ultrasonic water bath to ensure total extraction and homogenization, then placed back on dry ice. Samples were then centrifuged in an Eppendorf 5424 Centrifuge (Macquarie Park, New South Wales, Australia) at 5500 rpm for 15 min at room temperature. The supernatant was placed into either a glass insert or a clean microcentrifuge tube (depending on the biomass of the sample) and dried down overnight under an air pump in a fume hood. The substrate-only experiment had a greater tissue biomass, compared to the copper exposure experiment, so a glass insert was used to concentrate the sample.

The dried extracts were derivatized using either 30 µL (substrate-only exposure) or 20 µL (copper substrate exposure) of Methoxyamine in pyridine solution, (20 mg/mL). The samples were then vortexed for 30 s and left for 17 h at room temperature in the fume hood. Next, 30 µL (substrate- only exposure) or 20 µL (copper substrate exposure) of N-Methyl-N-(trimethylsilyl)trifluoroacetamide (MSTFA) were added to each sample, vortexed and left for 1 h. Analytical grade hexane was then added to each sample (600 µL for substrate- only exposure; 300 µL for copper substrate exposure) to perform GC–MS analysis. A pooled biological quality control (PBQC) was prepared by pooling 37.5 µL of extract from each sample mixing thoroughly and aliquoted into five replicates. PBQCs were analysed along with the samples to assess repeatability, instrument drift and quality control.

### Gas chromatography-mass spectrometry

The GC–MS analysis was performed using an Agilent 7890B gas chromatograph coupled to an Agilent 5977B mass spectrometer. The gas chromatograph was operated with a purge flow to the split vent at 2 min. The separation was performed with a DB5-MS column (30 m × 250 μm, 0.25 μm) using helium as carrier gas at a flow rate of 1 mL per min. The injection volume was 1μL, the injector temperature was 250 °C. The oven temperature program rose from 35 to 300 °C at 25 °C·min^−1^, held at this temperature for 5 min then raised to 310 °C (at 5 °C· min^−1^) and held for 5 min. Mass spectra were recorded at 1.5 scans/s over an m/z range of 35–550. Samples were analysed separately and in random order.

Metabolites were putatively annotated as level 2 compounds according to the chemical reporting standards in Sumner et al. (2007) (based upon spectral similarity with public/commercial spectral libraries) where possible, using the NIST library metabolite standards as part of an in-house database for identification. The remaining compounds were either identified to level 3 or 4. Level 3 IDs were based upon characteristic physicochemical properties of a chemical class of compounds, or by spectral similarity to known compounds of a chemical class. Level 4 IDs mean the compounds were unknown but although unidentified or unclassified these metabolites can still be differentiated and quantified based upon spectral data. Raw area retention times (RT) from each unidentified peak that significantly responded are listed in supplementary material (Table S5 and S6). Information regarding environmental metabolomic reporting standards as described by Morrison et al. (2007).

### Data analysis

Survival significance was determined using *t*-test: paired two sample for means between initial and surviving number of amphipods for each substrate. Reproduction was compared across all treatments using an analysis of variance (ANOVA)—two-factor with replication (the data conformed to the assumptions of ANOVA). Survival following the copper exposure, used a two tailed *t*-test between copper exposed and controls for each treatment.

A combination of multivariate and univariate statistical analysis was conducted as complete strategy for identification and selection of potential biomarkers, using the total area of abundance for each metabolite, as described in Lacalle-Bergeron et al. (2020). Metabolite values were normalized to the median value of each treatment to better account for unwanted system variation when dealing with metabolite extractions from whole organisms (Sinclair et al., 2019a, b). Values were then transformed (log_natural_) to reduce variance between metabolite abundance.

Multivariate statistical analysis was applied as a first step for interrogating the data in order to observe trends, grouping and/or outliers (Lacalle-Bergeron et al., 2020). As large data matrices were obtained, statistical tools, such as multivariate analysis, were necessary to reduce the data complexity and reveal underlying trends in featured metabolites. This is standard practice in Metabolomics. Principal component analysis (PCA) was initially run for unsupervised visualisation of metabolomic separation (supplementary material, fig S1 and S2). Partial least squares discriminate analysis (PLS-DA) (with appropriate validation) is a common method for determining separation in metabolomic data sets due to treatments when biological natural variation outweighs treatment induced variation (Blasco et al., 2015; Worley & Powers, 2013). PLS-DA was carried out using online MetaboAnalyst software (version 5.0), using both adult, juvenile and copper data sets to visualize overall separation of treatments (Pang et al., 2021). Model overview validation was performed via MetaboAnalyst using *Q*^2^ as an estimate for the predictive ability of the model, calculated via cross-validation (CV). In each CV, the predicted data was compared with the original data, and the sum of squared errors was calculated. The prediction error was then summed over all samples (Predicted Residual Sum of Squares or PRESS) (supplementary material, model overview of PLS-DA figs. S3- S7). Good predictions will have low PRESS or high *Q*^2^. The PRESS is a form of cross-validation used in regression analysis to provide a summary measure of the fit of a model to a sample of observations that were not themselves used to estimate the model. It is calculated as the sums of squares of the prediction residuals for those observations. For more details, please see to Szymańska et al. (2012)

An ANOVA was used to determine significant differences between substrates (*P* < 0.05). Following this, Tukeys post hoc test determined significance between treatments for each substrate (*p* < 0.05). Metabolites were analysed using one-factor ANOVA with treatment as the main effect with R Version 3.0.3 (R Core Team 2020). Each *p*-value was adjusted for false discovery rate using the Benjamini-Hochberg (BH) method for 5% to determine which metabolites responded significantly to treatments. Pathway analysis was carried for significant metabolites using online MetaboAnalyst. Reports for the analysis are presented in the supplementary material for adult amphipods, juvenile amphipods, Cellulose, Gauze, and Toilet paper.

## Results and discussion

### Substrate only exposure—adult and juvenile amphipods

#### Survival and reproduction

Water quality remained consistent, with dissolved oxygen > 80% and pH being 7–8. Conductivity (uS/cm) and ammonia levels (ppm) did not vary (supplementary material, Table S1).

**Fig. 1:**
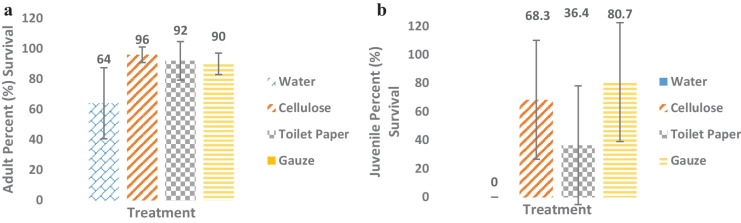
**a** Mean survival of adult amphipods in three substrates (cellulose, toilet paper and gauze) and a negative control of water only. **b** Survival of juvenile amphipods in substrates (gauze, toilet paper and cellulose). There was no survival in the water only treatments for juveniles. *N* = 5 replicates per treatment. Error bars show standard error of the mean

There was a 36% reduction in adults’ survival after the water-only exposure (e.g., no substrate at all). The initial mean and standard deviation was the same for each treatment (*M* = *10, SD* = *0).* Water had a decline in survival post exposure *(M* = *6.4, SD* = *2.8)* with a two tailed t-Test resulting with t_(4)_ = 4.812, *p* = 0.009. The survival of juveniles here was too low to continue the experiment and demonstrates that the amphipods needed a substrate to survive. There was a 5–10% decline in adults’ survival across all other substrates (Fig. 1a). It is important to note that for an ecotoxicology experiment to be valid, there needs to be at least 80% survival in controls which is why using a control with no substrate in this instance was not appropriate (Simpson & Kumar, 2016). Gauze had a decline in survival at the conclusion of the adult exposure *(M* = *9, SD* = *0.5)* with a t_(4)_ = 3.162, *p* = 0.034. Cellulose *(M* = *9.2, SD* = *1.7)* and toilet paper *(M* = *9.6, SD* = *0.3)* had higher survival post exposure with a t -Test result of t_(4)_ = 1.372, *p* = 0.242 and t_(4)_ = 1.633, *p* = 0.178, respectively. The surviving adult amphipods from the water only exposure were likely under stress from not having a substrate (which they would usually burrow into or potentially use as a food source or to hide from predators). If an ecotoxicology test was to not use substrate and introduce an additional stress, it would be impossible to tease out what the organism was responding to, lack of substrate or introduced stress. This further emphasizes that using appropriate material is essential to the experiment.

Survival in juveniles was more varied across the different substrates compared to adults (Fig. 1b). Toilet paper had large variation across replicates and an average decline in survival *(M* = *30, SD* = *704)*, averaging 36.4% survival post exposure with two tailed t-Test result of t_(4)_ = 5.816, *p* = 0.004. Cellulose had a higher average survival of 68% compared to toilet paper, *(M* = *70.6, SD* = *854.8)* with a *t*-test result of t_(4)_ = 2.423, *p* = 0.073. Gauze had the highest average survival in the juveniles 81%, *(M* = *103.8, SD* = *2337)* with t-Test result of t_(4)_ = 2.156, *p* = 0.097. These results indicate that different life stages of the same organisms have distinct preferences/needs regarding a suitable substrate. The difference between the substrates, could be the amphipods’ ability to cover themselves, hold on to and burrow into, the weight and texture preferences. Additionally, the differences could be affecting the overall biochemical functions of the amphipods which is what is explored further in this current paper.

**Fig. 2:**
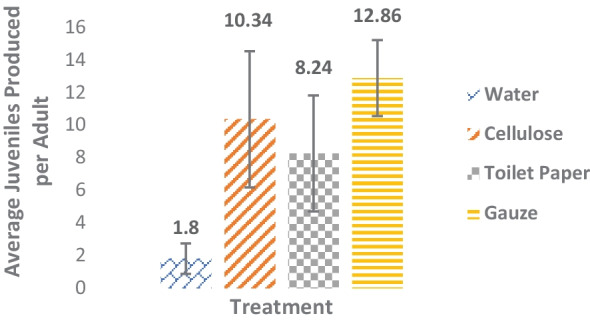
Average number of juvenile amphipods produced per adult in each substrate treatment. *N* = 5 replicates per treatment. Error bars show standard error of the mean

Reproduction as an endpoint was calculated by determining the ratio of juveniles produced per adult from each substrate (Fig. 2). With the greatest number of juveniles per adult produced from the gauze substrate 5:64. Cellulose had the second highest juveniles produced per adult with a ratio of adult: juvenile, 10:103. Toilet paper had a ratio of 5:41. Unsurprisingly, water having the lowest survival, also had the lowest reproduction ratio 5:9. ANOVA result F _(2,12)_ = 1.80, *p* = 0.21.

### Metabolites in adult and juvenile amphipods following substrate only exposure

**Fig. 3:**
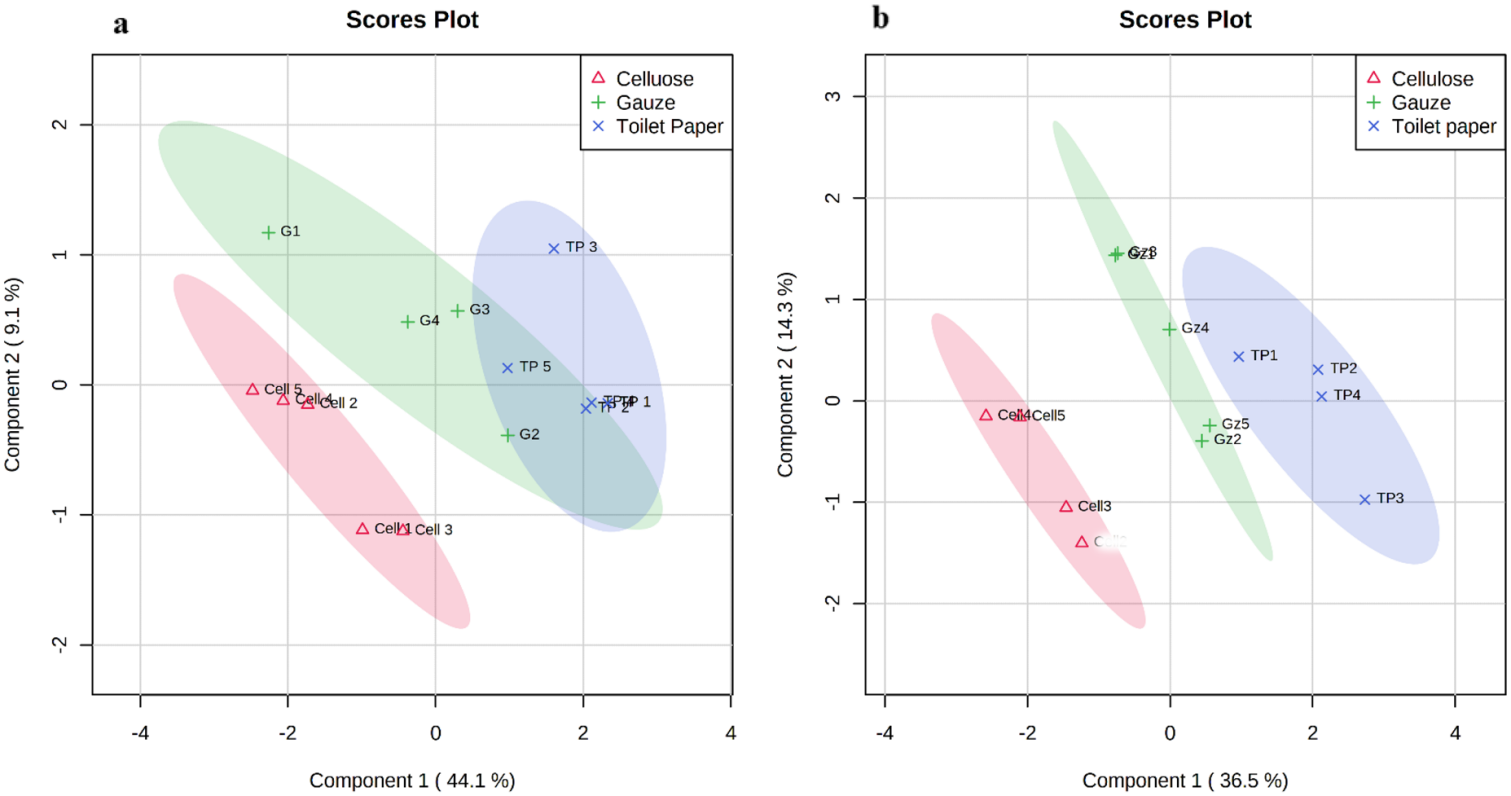
PLS-DA: separation of metabolites from adult (**a**) and juvenile (**b**) amphipods exposed to different substrates. PLS-DA Model overview validation can be found in the supplementary material (Figure S2 and S3)

There was separation in the overall metabolite profile in adult and juvenile amphipods exposed to the different substrates (Fig. 3a and b). This was particularly marked in the juvenile amphipods (Fig. 3b), where there was a clear separation between all three groups. This could be due to the extended length of exposure for the juveniles and/or due to their higher sensitivity to external conditions with respect to adults, and/or being in a major developmental stages of their life cycle (Vu et al., 2016). In contrast, there was less separation between the metabolic profiles of adult amphipods, particularly among the toilet paper and gauze substrate groups.

Univariate analysis of significant metabolite features was used to further elucidate the overall effects of the substrates on the amphipods. Substrate type had a significant effect on the abundance of several metabolites in both adult and juveniles. In adult amphipods, 20 metabolite features changed significantly in response to cellulose, toilet paper, gauze, and water-only treatments, out of a total of 80 that were detected (Table 1) including fatty and carboxylic acids. In contrast, in juveniles there were only 15 significantly different metabolite features that changed, from the three substrate treatments, out of a total of 66 features detected (Table 2), mostly amino acids.

**Table 1:** One-way analysis of variance for individual metabolites, measured following 14-day adult amphipods substrates exposure (gauze, cellulose, toilet paper and a water only negative control). Values listed are the significant differences of metabolite abundance between treatments (BH adjusted, *p* < 0.05). Using a 95% family-wise confidence level. Df (degrees of freedom) = .3/13 (treatments/total)

Metabolite	*F* value	ANOVA *p*-value	Comparison between substrates Tukeys post hoc *p* value		
			*Gz -Cell*	*TP-Cell*	*TP-Gz*
Tetradecanoic acid	41.31	0.000	0.000	0.000	0.765
Gluconic acid	38.29	0.000	0.000	0.725	0.000
Glucose	28.64	0.000	0.014	0.644	0.119
Succinic acid	24.39	0.000	0.342	0.000	0.000
Glutaric acid	17.94	0.000	0.301	0.000	0.008
Octadecanoic acid	11.59	0.001	0.243	0.002	0.061
Glyceryl monostearate	10.77	0.001	0.833	0.006	0.029
Glycine	10.27	0.001	0.849	0.001	0.004
Trehalose	10.04	0.001	0.997	0.500	0.391
Palmitic acid	9.269	0.002	0.005	0.003	0.990
Unidentified 3	9.245	0.002	0.215	0.017	0.482
Unidentified 1	8.474	0.002	0.886	0.015	0.056
Androsterone	8.065	0.003	0.398	0.002	0.032
Unidentified 2	7.416	0.004	0.948	0.041	0.015
Propanoic acid	7.253	0.004	0.372	0.932	0.703
Valine	6.474	0.006	0.935	0.011	0.031
Turanose	6.269	0.007	0.854	0.026	0.106
Valeric acid	5.404	0.012	0.286	0.374	0.016
Myo inositol	5.383	0.013	0.037	0.930	0.012
Elaidic acid	4.928	0.017	0.080	0.085	1.000

^1^Gz gauze; TP toilet paper; Cell cellulose; W water

^2^Unidentified metabolites, mass: charge ratio and retention time listed in supplementary material Table S5

**Table 2:** One-way analysis of variance for individual metabolites, measured following 28-day juvenile amphipods substrates exposure (gauze, cellulose, toilet paper). Values listed are the metabolites whose abundance differ significantly between treatments (BH adjusted, *p* < 0.05). Using a 95% family-wise confidence level. Df = .2/10 (treatments/total)

Metabolite		*F* value	ANOVA *P*-value	Comparison between substrates Tukeys post hoc *p* value		
				*Gz—Cell*	*TP -Cell*	*TP –Gz*
Octadecanoic acid		16.42	0.001	0.001	0.016	0.141
Glycerol		11.94	0.002	0.002	0.019	0.446
Palmitic acid		8.917	0.006	0.005	0.117	0.192
Proline		7.417	0.011	0.802	0.039	0.011
Alanine		7.21	0.012	0.910	0.033	0.013
Unidentified 3		6.813	0.016	0.027	0.026	1.000
Unidentified 7		6.167	0.018	0.014	0.277	0.225
Sucrose		6.905	0.018	0.019	0.916	0.098
Unidentified 1		6.113	0.018	0.029	0.030	0.988
Glutamic acid		6.849	0.023	0.912	0.096	0.023
Glutamine		5.545	0.024	0.064	0.026	0.754
Androst amine		5.358	0.026	0.022	0.452	0.186
Glucose		5.334	0.027	0.546	0.151	0.022
Talose		5.016	0.031	0.187	0.509	0.027
Valine		4.322	0.044	0.918	0.107	0.046

^1^*Gz* gauze; *TP* toilet paper; *Cell* cellulose

^2^List of unidentified metabolites, mass: charge ratio and retention time listed in supplementary material Table S6

Several significant metabolic features differed between adults and juveniles. For example, the adults had a greater number of fatty acids significantly change in concentration, where juveniles had more amino acids affected. However, some features were detected to be significant in both groups, such as mono-, di-, and poly saccharides. There were also metabolic differences associated with different substrates. Tetradecanoic (Myristic) acid was significantly reduced in the adult amphipods exposed to cellulose compared to the other treatments. Octadecanoic (Stearic) acid levels were significantly altered, however only between toilet paper cellulose in the adult exposure; cellulose compared to gauze and toilet paper in the juveniles. Toilet paper and cellulose had the greatest number of metabolites (13) change significantly in the adults, whereas, in the juveniles, the greatest number of metabolites (8) identified to respond was from the gauze and cellulose treatments (Table 2).

The extent of functions and pathways that have been altered due to the exposure of different substrates has been shown to be paramount when investigating the baseline responses in organisms. This can be through how the organisms use the substrates, e.g., to cover themselves, or burrow into and protect themselves, their metabolomic profiles are shifting. Thus, even if a control is present, when a contaminant is introduced the effects on the functions and pathways caused by the substrates could amplify or mask a contaminant response. Importantly, this can lead to misidentification of effect from a contaminant of interest depending on the substrates used. Thus, the change in abundance of these metabolites between treatments show that the amphipods are responding differently to the different substrates which is an important aspect that needs to be assessed when using metabolomics as an endpoint.

### Pathway analysis of adult and juvenile substrate only exposure

**Fig. 4:**
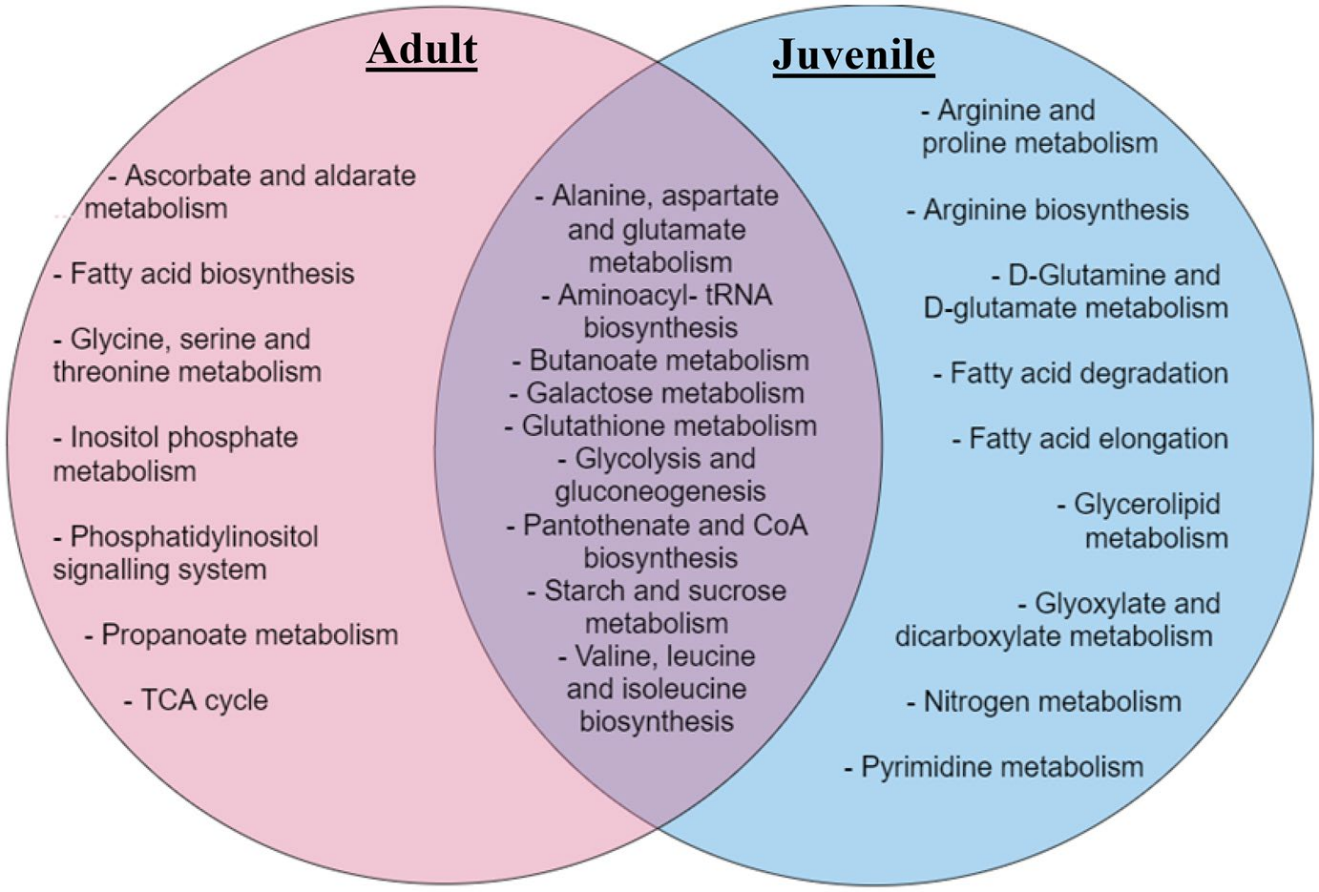
Venn diagram showing significant metabolite pathways that responded to the different substrate groups (cellulose, toilet paper and gauze), uniquely in adult amphipods and juvenile amphipods, as well as the pathways that changed in both life stages

The experimental observations can be placed into relevant biological context using pathway analysis tools (Karnovsky & Li, 2020). A pathway analysis was therefore carried out taking into consideration the significant metabolites that responded between the different life stages. This process is exploratory, and care must be taken to avoid over-interpretation of results. However, pathway identification is useful in generation of hypotheses surrounding observed metabolomic changes. In this case it identified seven pathways effected in the adult amphipods: nine in the juveniles and an additional nine pathways that responded across all three substrates (cellulose, toilet paper and gauze) in amphipods at both life stages (Fig. 4).

Many of the pathways detected to be affected in the adults feed into the tricarboxylic acid (TCA) cycle. The TCA cycle is used by organisms to generate energy for survival. It also provides precursors of certain amino acids that are used in numerous other reactions (Ryan et al., 2021). Its central importance to many biochemical pathways suggests that, once the overall health of the organism is disrupted, it can have much larger follow on impacts (Cavalcanti et al., 2014). Because of this, changes in TCA cycle metabolites tend to reflect a general metabolic disruption and are found in nearly all environmental metabolomics experiments and are thus not diagnostic. Propanoate metabolism is another intermediate of the TCA cycle that changed in response to only the different substrates, clearly indicating that after 14-days adult amphipods had a significant response to the various substrates. However again, this is not specific enough to be diagnostic of anything other than a general stress response.

Nine pathways responded to the different substrates in juveniles, including but not limited to, arginine and proline metabolism, which is one of the central pathways for the biosynthesis of the amino acids arginine and proline from glutamate (Patin et al., 2017). Glutamine and glutamate metabolism responded to the different substrates in the juveniles. Glutamine and glutamate metabolism can have an effect on ammonia homeostasis (Kelly & Stanley, 2001). Furthermore, there were nine pathways across both adults and juveniles that responded to the different substrates, such as alanine, aspartate, and glutamate metabolism; galactose metabolism; glutathione metabolism and glycolysis and gluconeogenesis. It is important to note that the total number of pathways affected by the choice of alternative substrates could have follow on effects for the health and functioning of the amphipods, no matter the stage of the organism’s life cycle.

Unknown metabolites deemed significant can often still be valuable to other researchers. Therefore, minimal requirements of reporting for unknown metabolites (retention time, prominent ion and fragment ion) has still been fulfilled in this current study (supplementary material, Table S5 and S6) (Lacalle-Bergeron et al., 2020; Sumner et al., 2007). Regarding the identified metabolites, certain pathways indicated activation and inhibition occurring in many sugar and lipid functions. These all are likely to feed into the overall fitness of the organism, potentially reducing its tolerance to other contaminants by weakening their energy reserves, as the overall survival indicated. Baseline environmental metabolomics can play a pivotal role in identifying biomarkers for environmental exposure. Therefore, these methodological decisions need to be assessed prior to running an exposure to understand what may be occurring before a contaminant is added. The data also show that metabolomics has a part to play in testing experimental design.

### Copper substrate exposure

#### Inductively coupled plasma mass spectrometry (ICP-MS)

The mean concentration of copper in the control water samples was 5 µg/L (± 0.2), and this was the background levels of copper measured in the RO water. The mean concentration of water in the exposures with copper added measured was 10 µg/L (± 1.3). Gauze treatment water had the lowest copper concentration at the conclusion of the experiment (8 µg/L), compared to cellulose (11 µg/L), and toilet paper (11 µg/L) (supplementary material, Table S4). Copper is naturally found in surface waters at low concentrations, usually < 10 µg/L (Department of Health and Human Services, 2019). Therefore, it was not surprising that copper was detected in the control water. This may also be the reason why higher concentrations of copper were measured (10 µg/L ± 1.3) in the treatments then the nominal concentrations.

**Fig. 5:**
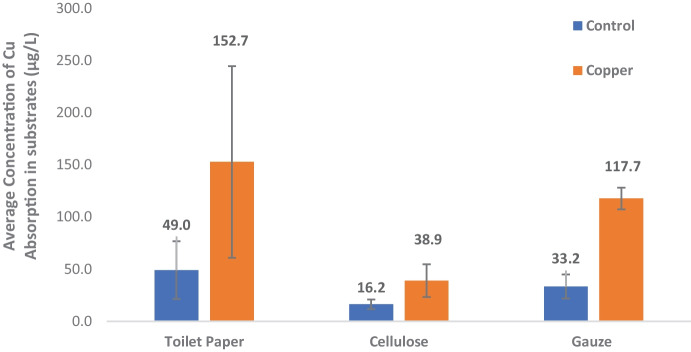
Mean concentration of Copper detected following 14-day exposure in control substrates (gauze, toilet paper and cellulose) and substrates from copper exposure using Inductively Coupled Plasma Mass Spectrometry (ICP-MS). *N* = 5 replicates per treatment. Error bars show standard error of the mean

Substrates accumulated higher concentrations of copper compared to the matched control, as anticipated (Fig. 4). The gauze control had an average copper concentration of 33 µg/kg (± 11.5), Cellulose controls absorbed less, 16 µg/kg (± 4.5) and the toilet paper control absorbed the most copper with a mean concentration of 49 µg/kg (± 27.8) (Fig. 5). Substrates had higher concentrations of copper, compared to control water and spiked water because the same substrates were used throughout the exposure whereas the stock water was changed after 7 days. Toilet paper absorbed the most copper out of the three substrates in both control and copper-exposed beakers and had the most variation across replicates (153 µg/kg ± 91.9) which makes the repeatability and reproducibility of copper exposure on amphipods difficult to control. Cellulose absorbed the least, in the control and treatments (33 µg/kg ± 15.7). Gauze on the other hand clearly accumulated more copper consistently across replicates then controls (183 µg/kg ± 10.4) (Fig. 5).

The bioavailability of copper depended on the substrate being used, potentially explains the difference in metabolic responses observed following copper exposure. The copper-spiked gauze treatments, accumulated copper, and resulted in the greatest number of metabolites that were significantly different to the unexposed matched controls. Amphipods tend to equally interact with substrates and the water column, so having copper bind to substrates uniformly across replicates is desirable for the best representation of sediment binding with heavy metals in an ecosystem.

### Survival

**Fig. 6:**
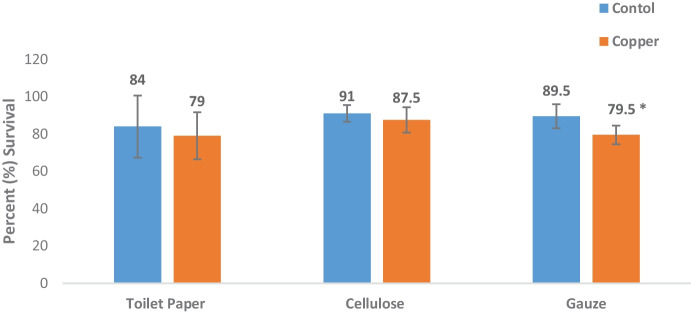
Mean survival of amphipods following 14-day exposure to control and copper substrates exposure. Error bars show standard error of the mean

Water quality remained consistent with dissolved oxygen > 80%; pH in the range of 7–8 and conductivity (1286- 1543 uS/cm) and ammonia levels (0–0.3 ppm) (supplementary material, Table S3). Survival of amphipods following the 14-days exposure to control substrates, was between 84 and 91%. There was a slight decrease of 5% survival in the toilet paper substrate after copper exposure and a decrease of 3.5% in the cellulose after copper treatment. Two-tailed *t*-test across substrates revealed survival of amphipods was only reduced when exposed to copper using a gauze substrate *(M* = *35.8, SD* = *6.7)*, t_(4)_ = 7.303, *p* = 0.002 (Fig. 6). This tells us that in terms of survival rate there is no difference between substrates as one might expect using standard measures. However, there was a clear metabolic difference as explained below. Toilet paper *(M* = *33.6, SD* = *39.3)*, t_(4)_ = 0.465, *p* = 0.66. Cellulose *(M* = *36.4, SD* = *3.3)*, t_(4)_ = 0.800, *p* = 0.468.

### Change in metabolite abundance of amphipods following copper substrate exposure

**Fig. 7:**
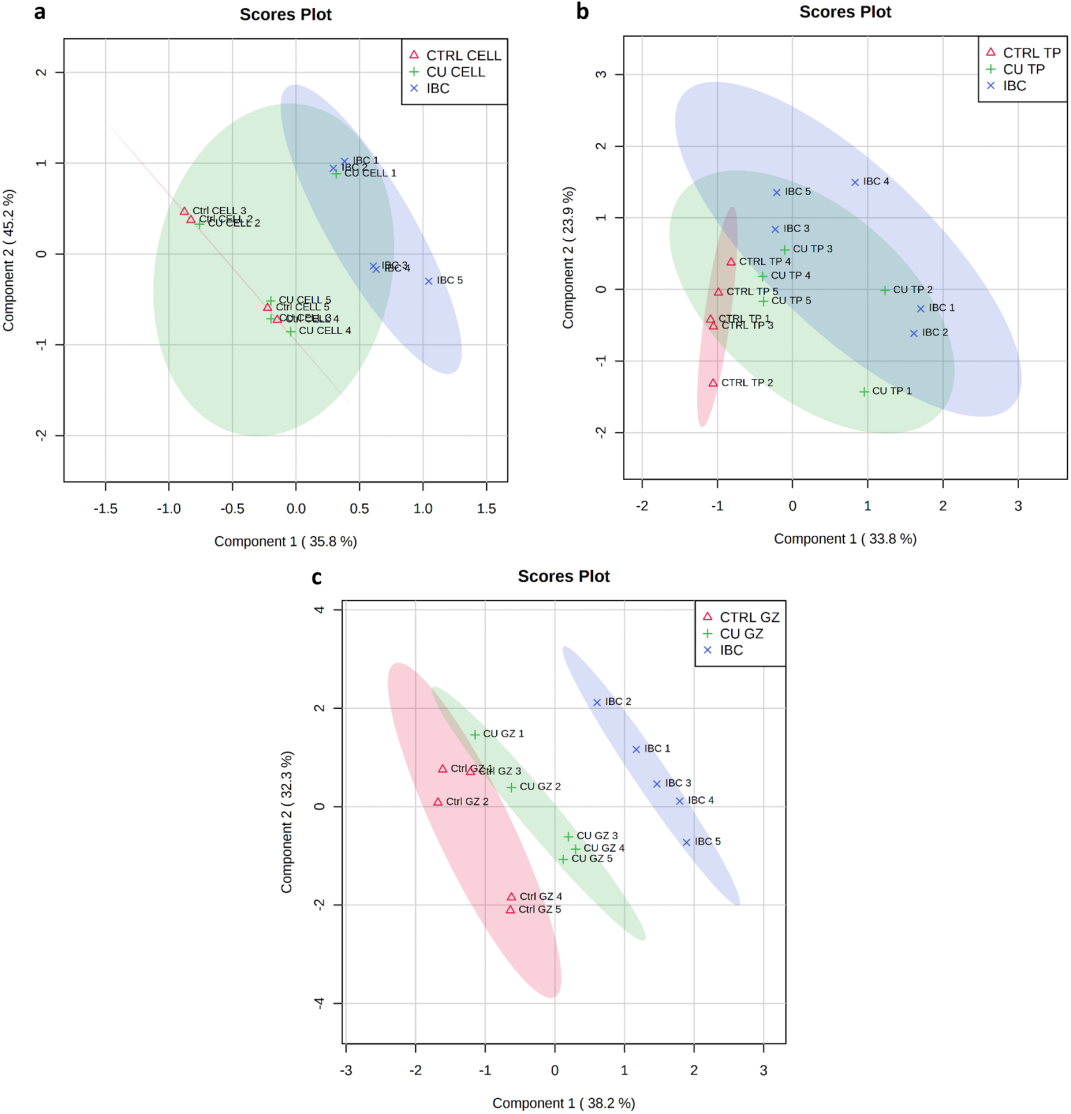
PLS-DA of metabolites extracted from amphipods exposed to copper for 14 days using cellulose (**a**) toilet paper (**b**) or gauze (**c**) as substrate controls and treated with copper following GC–MS. Initial baseline Controls (IBC) in each figure were collected at the commencement of the exposure period (day 0). PLS-DA Model overview can be found in the supplementary material (Figure S4–S6)

There was little separation between metabolites from day-0 initial baseline control (IBC) amphipods (collected at the commencement of the experiment), and amphipods exposed to Cu with the cellulose and toilet paper substrates (Fig. 7a and b). However, there was a distinct separation between treatments when using gauze as the substrate (Fig. 7c). The PLS-DA indicated that gauze induced a larger metabolic response to copper then cellulose.

There was a difference in amphipod biochemical responses with respect to copper exposure, depending on the substrate used. Changes between the IBC (14 days old) and exposure amphipods (28 days old) could potentially be due to the differences in ages of the amphipods, for example gauze indicated potential developmental changes from the IBC. Moreover, the lack of changes, could possibly be developmental effects on the amphipods caused by the choice of substrates, for example the limited changes between copper cellulose and the IBC; and copper toilet paper and the IBC. This would seem to indicate that substrate is a similar factor in the experiment to the Cu that can affect results, rather than something that has no affect at all. This means that substate choice may be as important to control for as more well- known confounders such as temperature or food. This is an important point for ecotoxicology in general.

### Univariate analysis of amphipods following copper substrate exposure

Exposure to copper were used to determine whether there was increased tolerance/sensitivity in the amphipods due to the choice of substrate. Survival remained relatively consistent across the three substrate treatment groups, suggesting survival was not impacted by the substrate type. Metabolomics analyses provided a more in-depth insight into the varying effects of the substrates on the amphipods, when exposed to copper. The abundance of 13 metabolites changed significantly in IBC groups compared to copper spiked substrates. Many of these metabolites showed a significant response between IBC and substrate, regardless of the substrate being spiked with copper.

As an example, there were six metabolite features that differed significantly in the cellulose treatment; however only two metabolites differed between unexposed and copper-spiked amphipods: galactopyranoside, a contributor to the galactose metabolism and linolenic acid, an essential fatty acid (Table 3). Galactopyranoside increased in abundance with cellulose as the substrate but remained at a constant level to the IBC amphipods when exposed to copper. In both cellulose and gauze treatment groups, linolenic acid increased in abundance compared to IBC. The remaining four metabolites from the cellulose treatment, were significantly changed between the IBC and cellulose, and not effected by the addition of copper. Cellulose had little difference in metabolite response to copper; potentially due to the lack of copper accumulation in the substrate, changing the copper availability to the amphipods (which actively interact with substrates) compared to gauze and toilet paper.

**Table 3:** One-way analysis of variance for individual metabolites, measured following 14-day amphipod copper spiked cellulose substrate. Values listed are the significant differences of metabolite for treatments (*p* < 0.05) and between treatments, including IBC amphipods (BH adjusted, *p* < 0.05). Using a 95% family-wise confidence level. Df = .2/11 (treatments/total)

Metabolite	*F* value	ANOVA *P*-value	Comparison between Cellulose and copper spiked cellulose amphipods Tukeys post hoc p value		
			*Cell – IBC*	*Cell – Cu Cell*	*Cu Cell – IBC*
Glycerol	30.64	0.000	∧ 0.000	0.747	∧ 0.000
Trehalose	15.81	0.001	∧ 0.001	0.352	∧ 0.005
Galactopyranoside	8.372	0.006	∧ 0.009	0.012	0.989
Lactic acid	6.893	0.012	∧ 0.159	0.332	∧ 0.009
Linolenic acid	5.905	0.018	∧ 0.019	0.052	∧ 0.806
Eicosenoic acid	4.776	0.032	∧ 0.135	0.760	∧ 0.031

∨Decreased in abundance compared to Initial Baseline Control (IBC) amphipods

∧Increased in abundance compared to Initial Baseline Control (IBC) amphipods

There were five metabolite features that significantly changed across in the toilet paper experiment, however four of these were between IBC and the substrate treatment groups (unexposed and exposed to copper). Only one metabolite, glycine, was significantly altered between copper-exposed and unexposed (Table 4). Glycine abundance increased by an average of 50% in both copper exposed toilet paper and gauze compared to the IBC amphipods.

**Table 4:** One-way analysis of variance for individual metabolites, measured following 14-day amphipod copper spiked Toilet Paper substrate. Values listed are the significant differences of metabolite for treatments (*p* < 0.05) and between treatments, including IBC amphipods (BH adjusted, *p* < 0.05). Using a 95% family-wise confidence level. Df = . 2/12 (treatments/total)

Metabolite	*F* value	ANOVA *P*-value	Comparison between Toilet Paper and copper spiked Toilet Paper amphipods Tukey’s post hoc *p* value		
			*TP – IBC*	*TP – Cu TP*	*Cu TP – IBC*
Glycerol	51.24	0.000	∧ 0.000	0.811	∧ 0.000
Lactic acid	49.62	0.000	∧ 0.000	0.857	∧ 0.000
Palmitic acid	29.51	0.000	∨ 0.000	0.069	∨ 0.001
Glycine	14.26	0.001	∧ 0.647	0.004	∧ 0.001
Eicosenoic acid	5.042	0.026	∨ 0.044	1.000	∨ 0.043

∨Decreased in abundance compared to Initial Baseline Control (IBC) amphipods

∧Increased in abundance compared to Initial Baseline Control (IBC) amphipods

Interestingly, gauze had the greatest number of metabolites that differed significantly, with 13 metabolites (Table 5), compared to cellulose that had six metabolites and toilet paper which had five metabolites out of a total of 31 features. The differences between control and treatment groups (substrate and copper spiked substrate), was particularly clear in the gauze treatment. Seven metabolites responded significantly between copper-spiked and unexposed amphipods when using gauze as a substrate: e.g., glucose, propanoic acid, succinate acid, palmitic acid, tetradecanoic acid, isopropanol, and lactic acid. In gauze, galactopyranoside levels increased when exposed to copper and decreased in the amphipods from the unexposed treatment.

**Table 5:** One-way analysis of variance for individual metabolites, measured following 14-day amphipod copper spiked Gauze substrate. Values listed are the significant differences of metabolite for treatments (*p* < 0.05) and between treatments, including IBC amphipods (BH adjusted, *p* < 0.05). Using a 95% family-wise confidence level. Df = . 2/12 (treatments/total)

Metabolite	*F* value	ANOVA *P* value	Comparison between Gauze and copper spiked Gauze amphipods Tukey’s post hoc *p* value		
			*Gz – IBC*	*Gz – Cu Gz*	*Cu Gz – IBC*
Glycerol	156.7	0.000	∧ 0.000	0.773	∧ 0.000
Glucose	43.47	0.000	∧ 0.000	0.020	∧ 0.000
Glycine	41.44	0.000	∨ 0.444	0.000	∧ 0.000
Propanoic acid	41.4	0.000	0.983	0.000	∧ 0.000
Succinate acid	28.51	0.000	∨ 0.196	0.000	∧ 0.000
Palmitic acid	20.1	0.000	∨ 0.000	0.003	∧ 0.142
Tetradecanoic acid	13.66	0.001	1.000	0.002	∨ 0.002
Galactopyranoside	12.83	0.001	∨ 0.352	0.001	∧ 0.012
Isopropanol	11.07	0.002	∨ 0.333	0.002	∧ 0.022
Linolenic acid	9.704	0.003	∧ 0.667	0.016	∧ 0.003
Lactic acid	8.471	0.005	∨ 0.360	0.053	∧ 0.004
Trehalose	7.564	0.007	∧ 0.092	0.303	∧ 0.006
Eicosenoic acid	4.525	0.034	∨ 0.027	0.338	∧ 0.309

∨Decreased in abundance compared to Initial Baseline Control (IBC) amphipods

∧Increased in abundance compared to Initial Baseline Control (IBC) amphipods

Cellulose, toilet paper, and gauze without the addition of copper resulted in changes to different metabolites compared to IBC; they also altered the abundance of the metabolites in different ways, i.e., some increased in abundance and some decreased compared to the IBC and unexposed, e.g., galactopyranoside.

### Pathway analysis of amphipod from different substrates exposed to copper

**Fig. 8:**
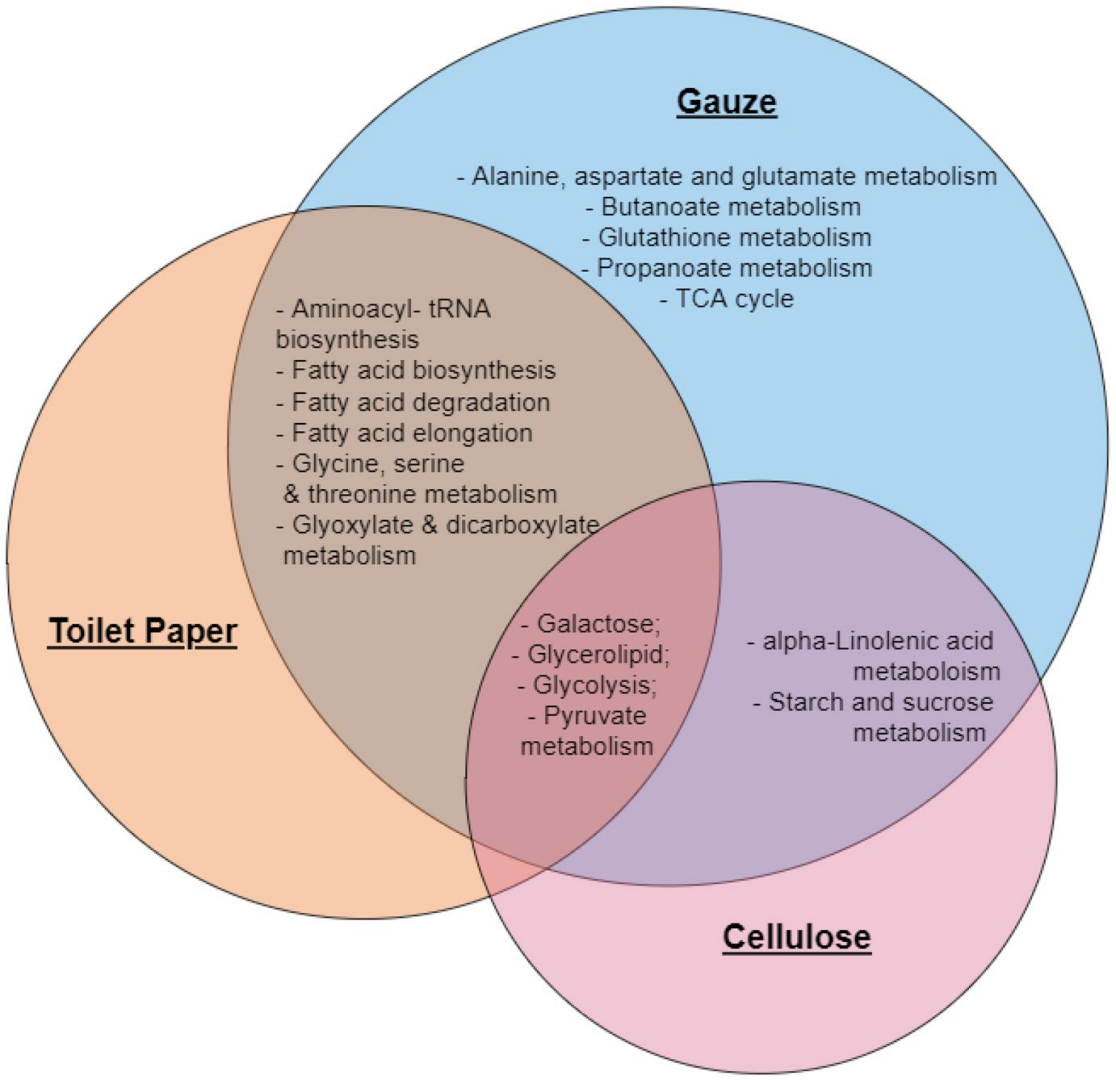
Venn diagram showing significant metabolite pathways that changed in response to copper in each of the substrate treatments (cellulose, toilet paper and gauze)

Four pathways changed significantly in response to copper, irrespective of substrate: galactose metabolism, an important component of glycolipids and glycoproteins (Cohn & Segal, 1973); glycolysis/gluconeogenesis, which converts glucose to pyruvate prior to entering the TCA cycle; glycerolipid metabolism, necessary for membrane formation, caloric storage and crucial for intracellular signaling processes (Voelker, 2013), and pyruvate metabolism contributing to the TCA cycle (Fig. 8).

Interestingly, when gauze was used as a substrate, an additional six metabolite pathways were affected by copper exposure, including alanine, aspartate and glutamate metabolism, butanoate metabolism, glutathione metabolism, propanoate metabolism and the tricarboxylic acid (TCA) cycle. These pathways were not impacted when toilet paper or cellulose were used as substrates.

Toilet paper and cellulose affected different pathways. For example, alpha-linolenic acid metabolism, essential to metabolism of fatty acids; and starch and sucrose metabolism, responsible for the conversion of disaccharides to monosaccharides were shown to be affected by both cellulose and gauze treatments. Whereas, toilet paper and gauze affected six pathways; fatty acid biosynthesis, degradation and elongation; glycine, serine and threonine metabolism, that aids in protein balance and immune reactions in organisms; glyoxylate and dicarboxylate metabolism, characterized by a variety of reactions involving the two metabolites that contribute to the TCA cycle (Li et al., 2020); and amino acyl-tRNA biosynthesis that is pivotal to determine how the genetic code is interpreted as amino acids (Ling et al., 2009) (Fig. 8).

Gauze resulted in an additional five pathways not detected in response to the other substrates, including the TCA cycle; alanine, aspartate and glutamate metabolism as well as, butanoate glutathione and propanoate pathways.

Measuring individual metabolite concentrations and identifying significant variations reflecting the biological function or regulation within an organism can identify metabolic pathways potentially affected by contaminants. It is also useful in generation of hypotheses surrounding metabolic changes for further investigation under controlled conditions (Taylor et al., 2019). It is therefore very important to ensure that the metabolites that are changing are only changing in response to the compound or compounds under study.

### Choice of substrate for ecotoxicology exposures

The list of metabolite pathways, associated with the significant metabolites that responded to copper substrate exposure, highlighted several similarities to the substrate only exposure. This was relatively surprising, as the expectation was that detox/oxidative stress responses would be underway in the organisms following copper exposure only. Potentially the amphipods were responding to the substrates foremost and would eventually start a detox/oxidative stress response; an exposure over a longer-period of time could determine this. Amphipods exposed to copper tended to show a more sensitive response when using gauze as a substrate, as suggested by the number of significantly responding metabolites to that treatment. Additionally, gauze substrate resulted in better differentiation between copper-exposed and unexposed amphipods.

It is important to consider in ecotoxicological exposures how the substrate may impact the variations (number of significant metabolites, changes in direction of abundance (i.e., increased/decreased) and extent of the variation—fold change) and may hide the possible response that is under investigation. Additionally investigate data processing applications with the potential to use the isometric log-ratio (irl) approach to further elucidate more reliable results with increased effect sizes (Lehmann, 2015). Any alternative substrate for aquatic ecotoxicology assessments needs to not only be optimal for the organisms but also be easy to use and to provide repeatability of results. Gauze from the sterilized bandaged provided repeatability of results as it did not degrade over the period of exposure and most importantly had the highest survival rate. Therefore, gauze was the most user friendly and optimal substrate for experimental repeatability. This study aimed to investigate substrates effects and narrowed the window for further targeted explorations. Future work can determine the range of parameters that may increase or decrease an organism’s stress response to contaminant exposure, such as determining the effect of sampling from an established culture, the pack down methodology, feeding regimes, and light and temperature exposure (Rosenblum et al., 2006; Shi et al., 2012).

## Conclusion

Ecotoxicological experiments are useful tools to understand the effects of exposure to contaminants on terrestrial and aquatic organisms and they generally provide data to derive guideline values for the protection of species in the environment. This work found that the choice of substrate (gauze, toilet paper, and cellulose) had an impact on metabolomic responses in amphipods at different life stages and following exposure to copper even though it did not affect survival. This would seem to indicate that substrate is a similar factor in the experiment to the Cu that can affect the results, rather than something that has no affect and thus also demonstrates the utility of metabolomics to pick up changes that traditional tests might miss. Substate choice may be as important to control for as more well-known confounders such as temperature or food. This is an important point for ecotoxicology in general. The current research found that gauze was an appropriate substrate to use for ecotoxicology experiments using amphipods as target organisms due to its stability across replicate response and ease of use. The amphipods had a more consistent metabolomic response using gauze, with a higher reproduction rate, leading to a better baseline for experimental exposure before adding contaminants. This increases our overall confidence in the results, which can be beneficial when investigating metabolomic or other sublethal biochemical endpoints. This information can add value to our overall understanding of causal relationships between exposure and contaminants in the ecosystems or potentially linked to human health. Results from such experiments can carry weight in the way experiments are conducted or how environments are remediated, therefore it is essential to have a valid and trustable connection between the identified effects and the exposure. The current study ultimately demonstrates the need to ensure the methodology that has been widely adopted and used for many years, such as the choice of substrates in ecotoxicological experiments, is still appropriate for the sensitive markers of exposure we are in search of today.

## Supplementary Information

Below is the link to the electronic supplementary material.

Supplementary file1 (DOCX 1440 KB)

## Data Availability

Data is available in Supplementary martial. Raw data/ additional data available upon request from the corresponding author.

## Declarations

### Plant reproducibility

Not applicable (N/A) for this research.
