# Supplementary material for: Is substrate choice an overlooked variable in ecotoxicology experiments?
Source: Environ Monit Assess. 2023 Jan 30;195(2):344. doi: 10.1007/s10661-023-10935-1 (PMC9886613; doi:10.1007/s10661-023-10935-1)
Supplement: Supplementary file 1 — Supplementary file1 (DOCX 1440 KB) [file 10661_2023_10935_MOESM1_ESM.docx]

Supplementary material

**Is Substrate Choice an Overlooked Variable in Ecotoxicology Experiments?**

Georgia M. Sinclair ^1^, Michela Di Giannantonio^2^, Oliver A.H. Jones ^1^, and Sara M. Long ^3^

^1^ Australian Centre for Research on Separation Science (ACROSS), School of Science, RMIT University, Bundoora West Campus, PO Box 71, Bundoora, VIC 3083, Australia

^2^ Institute for the study of Anthropic Impacts and Sustainability in Marine Environment – National Research Council (CNR-IAS), Genova Italy

^3^ Aquatic Environmental Stress (AQUEST) Research Group School of Science, RMIT University, Bundoora, VIC 3083, Australia

***** Author to whom correspondence may be addressed: s3762567@student.rmit.edu.au

Table S1. Weekly water quality for adult and juvenile amphipods life stages substrates exposure.

|  | **Weekly Water Quality** | | |  |  |  | **Notes:** | |
| --- | --- | --- | --- | --- | --- | --- | --- | --- |
| **31.08.2020** | | **DO**  **(%)** | **pH** | **Cond.**  **(uS/cm).** | **NH_4_ (ppm)** | **Temp.**  **(^o^C)** | Adults and juveniles | |
|  | Water | 104.3 | 8.26 | 1436 | 3 | 19.7 |  |  |
|  | Gauze | 100.6 | 8.06 | 1373 | 0 | 19.6 |  |  |
|  | TP | 95.3 | 7.98 | 1414 | 0 | 20.8 |  |  |
|  | Cel | 98.7 | 8 | 1436 | 0 | 20.7 |  |  |
| **7.09.2020** |  | **DO %** | **pH** | **Cond.** | **NH_4_** | **Temp.** |  |  |
|  | Water | 81.2 | 7.97 | 1487 | 4 | 20.5 |  |  |
|  | Gauze | 63.1 | 7.44 | 1435 | 0.25 | 21 |  |  |
|  | TP | 53.2 | 7.4 | 1466 | 0.25 | 20.4 |  |  |
|  | Cel | 77.5 | 7.87 | 1543 | 0.25 | 20.6 |  |  |
| **14.09.2020** | | **DO %** | **pH** | **Cond.** | **NH_4_** | **Temp.** | Juveniles only | |
|  | Water | 96.4 | 8.24 | 1324 | 1 | 19.1 | Water exposure Ended | |
|  | Gauze | 93.9 | 8.09 | 1286 | 0 | 19 |  |  |
|  | TP | 93.5 | 8.16 | 1298 | 0 | 19.1 |  |  |
|  | Cel | 95.9 | 8.18 | 1293 | 0 | 19 |  |  |
| **21.09.2020** | | **DO %** | **pH** | **Cond.** | **NH_4_** | **Temp.** |  |  |
|  | Water |  |  |  |  |  |  |  |
|  | Gauze | 100.2 | 8.27 | 1345 | 0.25 | 19.4 |  |  |
|  | TP | 93.6 | 8.07 | 1296 | 0.25 | 19.4 |  |  |
|  | Cel | 98.9 | 8.32 | 1336 | 0.25 | 19.4 |  |  |
| **28.09.2020** | | **DO %** | **pH** | **Cond.** | **NH_4_** | **Temp.** |  |  |
|  | Water |  |  |  |  |  |  |  |
|  | Gauze | 107.7 | 8.44 | 1311 | 0 | 18.9 |  |  |
|  | TP | 101.4 | 8.11 | 1360 | 0 | 18.7 |  |  |
|  | Cel | 96.4 | 8 | 1324 | 0 | 18.7 |  |  |

Table S2. Total Survival of post-gravid adults; initial count of juveniles before continuation of exposure and total survival following exposure.

| Survival | 7.09.2020 |  |  |  | 5.10.2020 |
| --- | --- | --- | --- | --- | --- |
|  | **Adult (10)** | **Juvenile** | **Juv. A** | **Juv. B** | **Total** |
| Water 1 | 6 | 5 | 0 | 0 | 0 |
| Water 2 | 9 | 20 | 0 | 0 | 0 |
| Water 3 | 7 | 30 | 0 | 0 | 0 |
| Water 4 | 5 | 10 | 0 | 0 | 0 |
| Water 5 | 5 | 25 | 0 | 0 | 0 |
| Gauze 1 | 10 | 167 | 91 | 76 | 167 |
| Gauze 2 | 8 | 115 | 35 | 35 | 70 |
| Gauze 3 | 9 | 144 | 73 | 68 | 141 |
| Gauze 4 | 9 | 106 | 40 | 48 | 88 |
| Gauze 5 | 9 | 111 | 22 | 31 | 53 |
| TP 1 | 10 | 128 | 22 | 25 | 47 |
| TP 2 | 7 | 30 |  | 5 | 5 |
| TP 3 | 10 | 116 | 50 | 17 | 67 |
| TP 4 | 10 | 72 |  | 23 | 23 |
| TP 5 | 9 | 66 |  | 8 | 8 |
| Cel 1 | 10 | 44 | 7 | 16 | 23 |
| Cel 2 | 10 | 156 | 22 | 53 | 75 |
| Cel 3 | 9 | 144 | 54 | 47 | 101 |
| Cel 4 | 9 | 76 | 44 | 25 | 69 |
| Cel 5 | 10 | 97 | 45 | 40 | 85 |

Table S3. Water quality for copper – substrates exposure at 7 days and 14 days.

| **25.06.21** |  | **DO**  **(%)** | **pH** | **Cond.**  **(uS/cm).** | **NH_4_ (ppm)** | **Temp.**  **(^o^C)** |
| --- | --- | --- | --- | --- | --- | --- |
|  | Gauze | 90.4 | 7.9 | 1373.0 | 0.0 | 19.7 |
|  | Cellulose | 83.3 | 8.1 | 1381.0 | 0.0 | 20.5 |
|  | Toilet Paper | 88.2 | 8.2 | 1392.0 | 0.0 | 20.9 |
|  | Cu Gauze | 88.4 | 8.2 | 1387.0 | 0.0 | 19.9 |
|  | Cu Cellulose | 88.0 | 8.2 | 1388.0 | 0.0 | 19.7 |
|  | Cu Toilet Paper | 86.9 | 8.1 | 1373.0 | 0.0 | 19.5 |
| **2.07.21** |  | **DO %** | **pH** | **Cond.** | **NH_4_** | **Temp.** |
|  | Gauze | 84.2 | 8.1 | 1343.0 | 0.3 | 20.4 |
|  | Cellulose | 83.5 | 8.2 | 1403.0 | 0.0 | 20.3 |
|  | Toilet Paper | 86.9 | 8.3 | 1419.0 | 0.3 | 20.6 |
|  | Cu Gauze | 86.8 | 8.3 | 1385.0 | 0.0 | 21.1 |
|  | Cu Cellulose | 83.4 | 8.2 | 1364.0 | 0.0 | 21.2 |
|  | Cu Toilet Paper | 87.2 | 8.3 | 1421.0 | 0.3 | 21.0 |

Table S4. Inductively Coupled Plasma Mass Spectrometry (ICP-MS). Water and substrates measurements.

| **Type** | **Group** | | **Sample** | | **Replicate** | **Conc. ppb** | **Conc. RSD** |
| --- | --- | --- | --- | --- | --- | --- | --- |
| Water | | Day 0 | | Copper Stock |  | 11.390 | 0.9 |
|  |  |  |  | Control |  | 4.854 | 1.9 |
|  |  | Day 14 | | Gauze | Copper | 8.164 | 1.9 |
|  |  |  |  | Gauze | Control | 5.044 | 0.3 |
|  |  |  |  | Cellulose | Copper | 10.648 | 1.9 |
|  |  |  |  | Cellulose | Control | 4.668 | 2.3 |
|  |  |  |  | Toilet Paper | Copper | 11.200 | 0.5 |
|  |  |  |  | Toilet Paper | Control | 5.114 | 1.8 |
| Substrates | | Copper | | Gauze | R1 | 120.108 | 1.3 |
|  |  |  |  | Gauze | R2 | 116.327 | 1.2 |
|  |  |  |  | Gauze | R3 | 107.789 | 0.9 |
|  |  |  |  | Gauze | R4 | 108.124 | 1.2 |
|  |  |  |  | Gauze | R5 | 136.361 | 0.2 |
|  |  | Control | | Gauze | R1 | 51.130 | 0.5 |
|  |  |  |  | Gauze | R2 | 28.829 | 1.9 |
|  |  |  |  | Gauze | R3 | 41.826 | 0.3 |
|  |  |  |  | Gauze | R4 | 23.882 | 3 |
|  |  |  |  | Gauze | R5 | 20.549 | 0.7 |
|  |  | Copper | | Cellulose | R1 | 23.034 | 0.7 |
|  |  |  |  | Cellulose | R2 | 39.304 | 1.5 |
|  |  |  |  | Cellulose | R3 | 39.939 | 1.5 |
|  |  |  |  | Cellulose | R4 | 53.161 | 1.2 |
|  |  | Control | | Cellulose | R1 | 7.720 | 1.8 |
|  |  |  |  | Cellulose | R2 | 19.710 | 1.5 |
|  |  |  |  | Cellulose | R3 | 15.821 | 0.9 |
|  |  |  |  | Cellulose | R4 | 20.407 | 0.9 |
|  |  |  |  | Cellulose | R5 | 17.272 | 0.5 |
|  |  | Copper | | Toilet Paper | R1 | 310.772 | 1.1 |
|  |  |  |  | Toilet Paper | R2 | 38.451 | 1.6 |
|  |  |  |  | Toilet Paper | R3 | 95.378 | 0.3 |
|  |  |  |  | Toilet Paper | R4 | 179.805 | 1.3 |
|  |  |  |  | Toilet Paper | R5 | 139.301 | 20.6 |
|  |  | Control | | Toilet Paper | R1 | 84.745 | 1.8 |
|  |  |  |  | Toilet Paper | R2 | 6.991 | 1 |
|  |  |  |  | Toilet Paper | R3 | 74.222 | 12.9 |
|  |  |  |  | Toilet Paper | R4 | 39.574 | 1.2 |
|  |  |  |  | Toilet Paper | R5 | 39.365 | 0.9 |

Raw area retention times (RT) from each peak for each treatment has been recorded.

Table S5. Adults unidentified metabolites.

| Unidentified 1  m/z 113; RT:8.93 | Unidentified 2  m/z 147; RT:9.95 | Unidentified 3  m/z 133; RT:16.728 |
| --- | --- | --- |

Table S6. Juveniles unidentified metabolites.

| Unidentified 1 | Unidentified 2 | Unidentified 3 | Unidentified 4 | Unidentified 5 |
| --- | --- | --- | --- | --- |
| m/z 147; RT:6.487 | m/z 147; RT:8.67 | m/z 128; RT: 8.922 | m/z 147;  RT:9.15 | m/z 147;  RT:9.39 |
| Unidentified 6 | Unidentified 7 | Unidentified 8 | Unidentified 9 | Unidentified 10 |
| m/z 173;  RT:9.939 | m/z 327; RT:11.756 | m/z 147; RT:11.916 | m/z 147;  RT:13.882 | m/z 133;  RT:16.671 |

**Principal Component Analysis (PCA)** was initially run for unsupervised visualisation of metabolomic separation for Adult and Juvenile Amphipods; as well for the copper substrate exposure (cellulose, toilet paper and gauze).

*Fig. S1 PCA: separation of metabolites from adult (a) and juvenile (b) amphipods exposed to different substrates.*


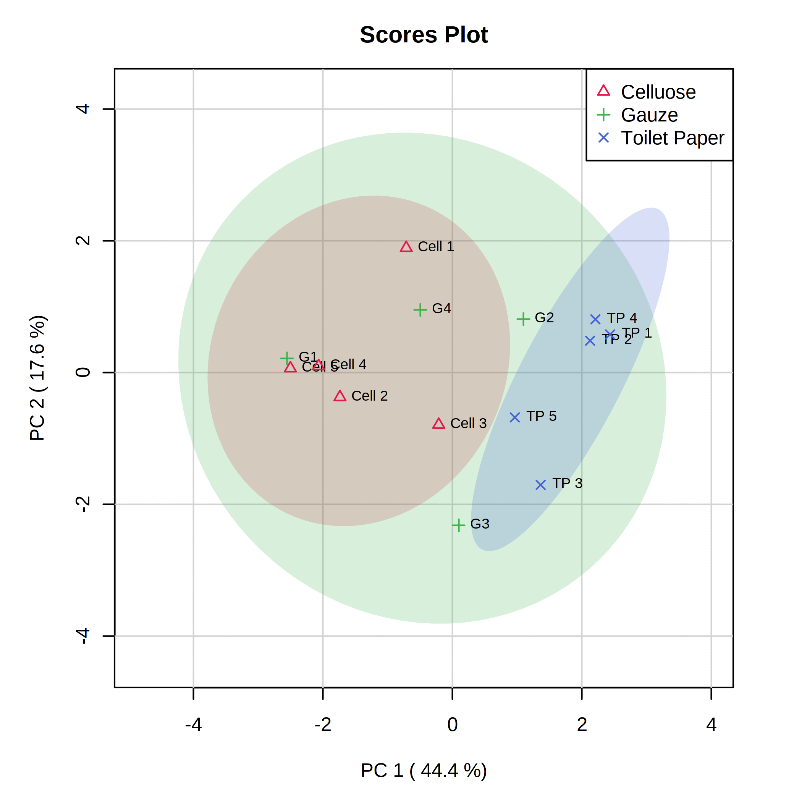

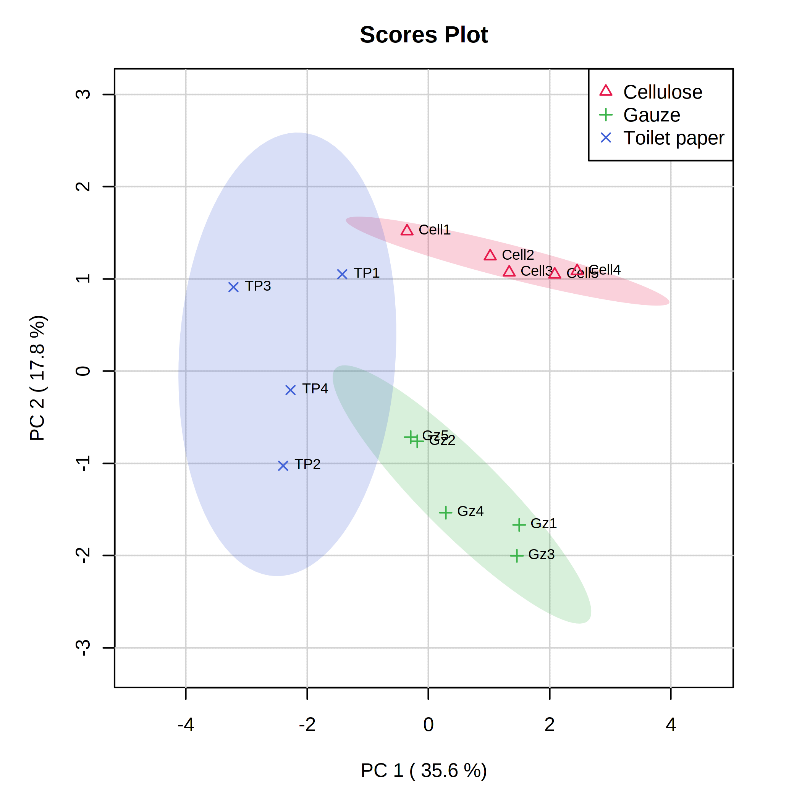

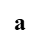

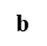


*Fig. S2. PCA of metabolites extracted from amphipods exposed to copper for 14 days using Cellulose (a) Toilet Paper (b) or Gauze (c) as substrate controls and treated with copper following GC-MS. Initial baseline Controls (IBC) in each figure were collected at the commencement of the exposure period (day 0).*


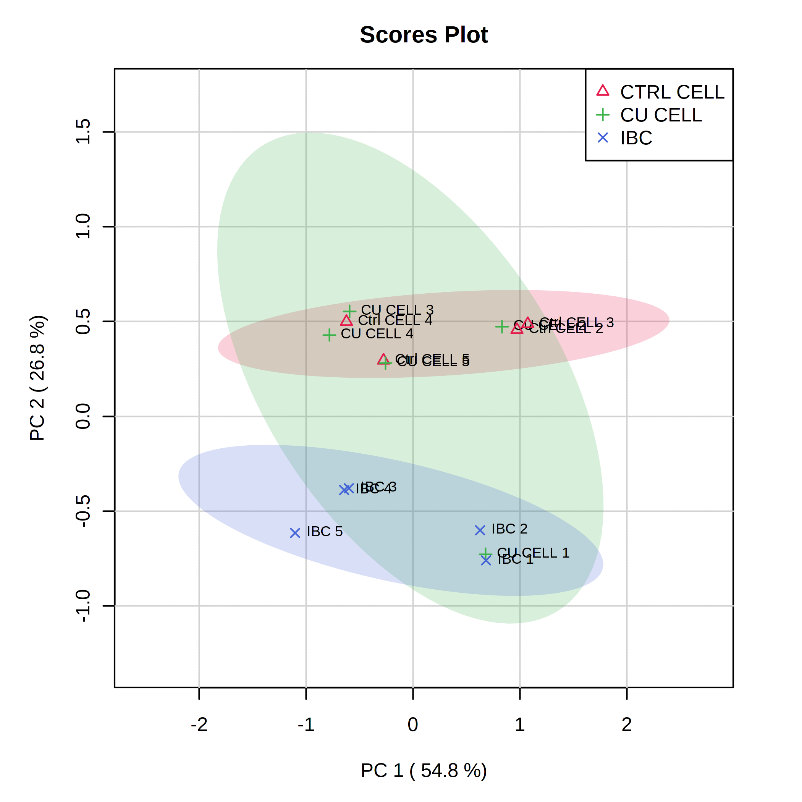

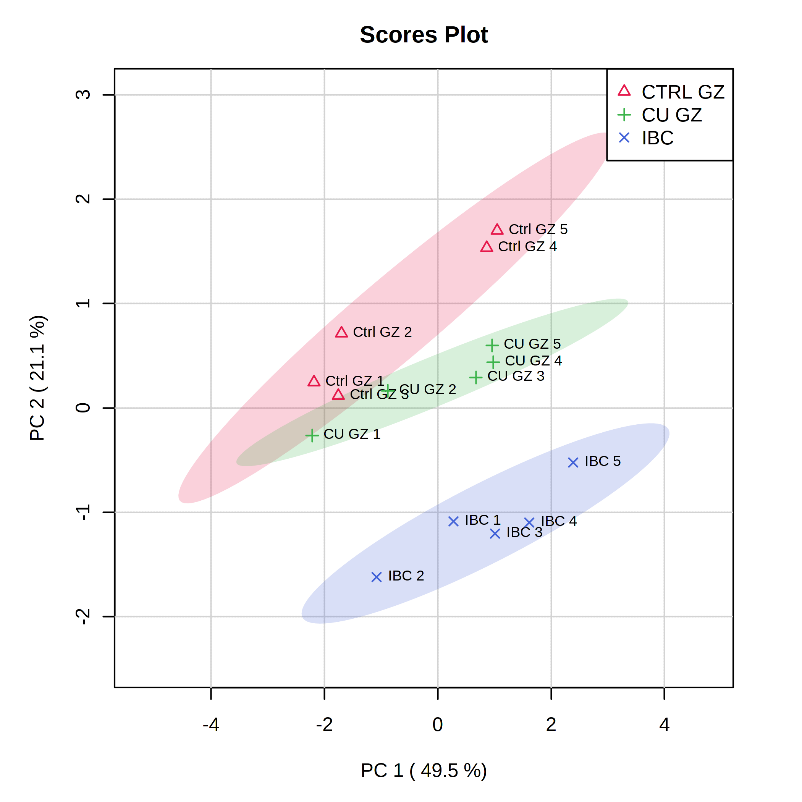

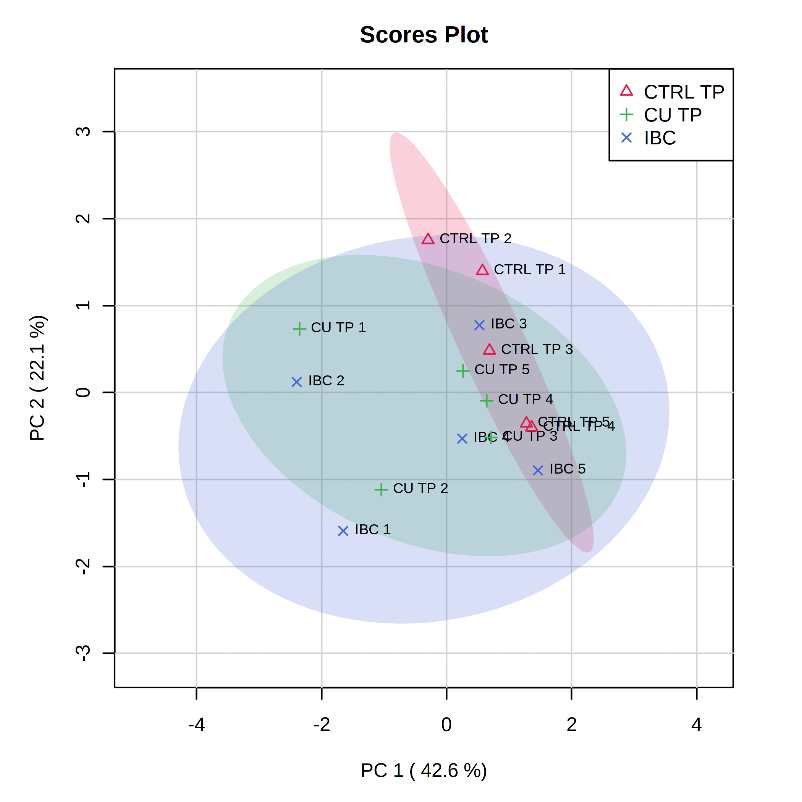

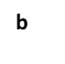

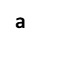

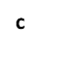


*
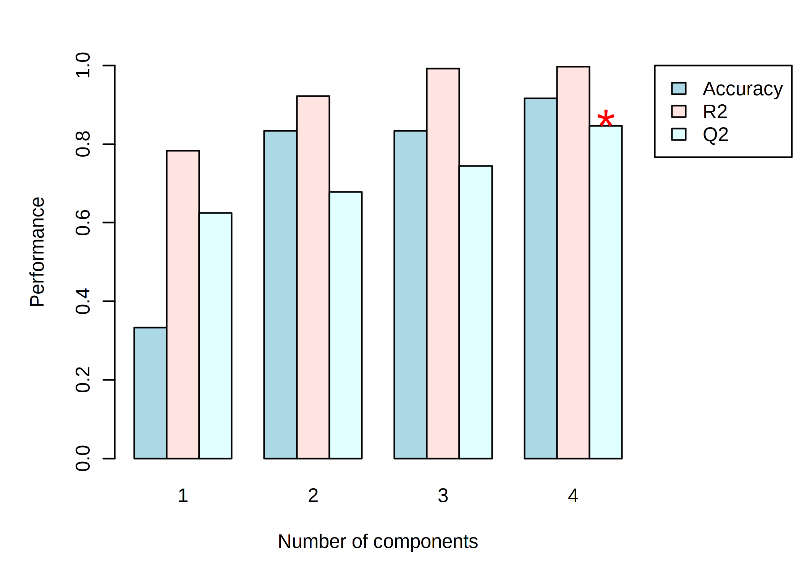
*

*Fig. S3. Partial Lease Squares - Discriminant Analysis (PLS-DA) – Adult’s separation across substrates model overview.*

*
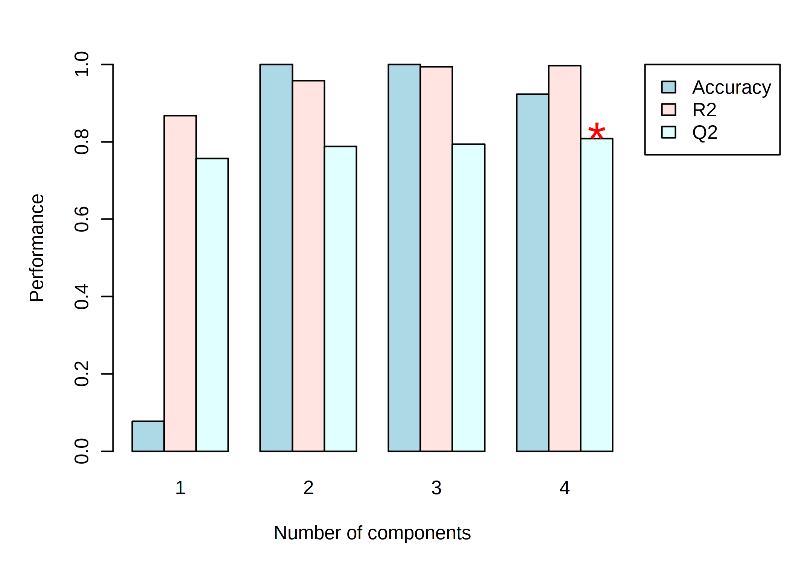
*

*Fig. S4. Partial Lease Squares - Discriminant Analysis (PLS-DA) – Juvenile’s separation across substrates model overview.*

*
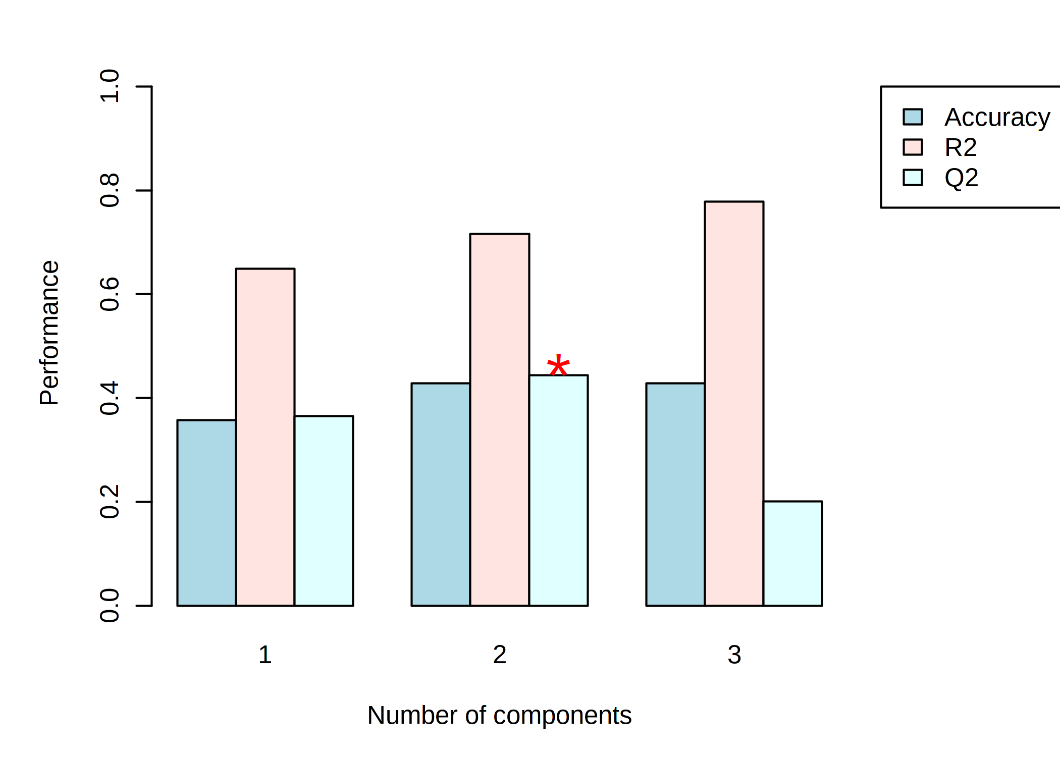
*

*Fig. S5. Partial Lease Squares - Discriminant Analysis (PLS-DA) Amphipods exposed and unexposed to copper in Cellulose (including Initial Baseline amphipods) separation across substrates model overview.*

*
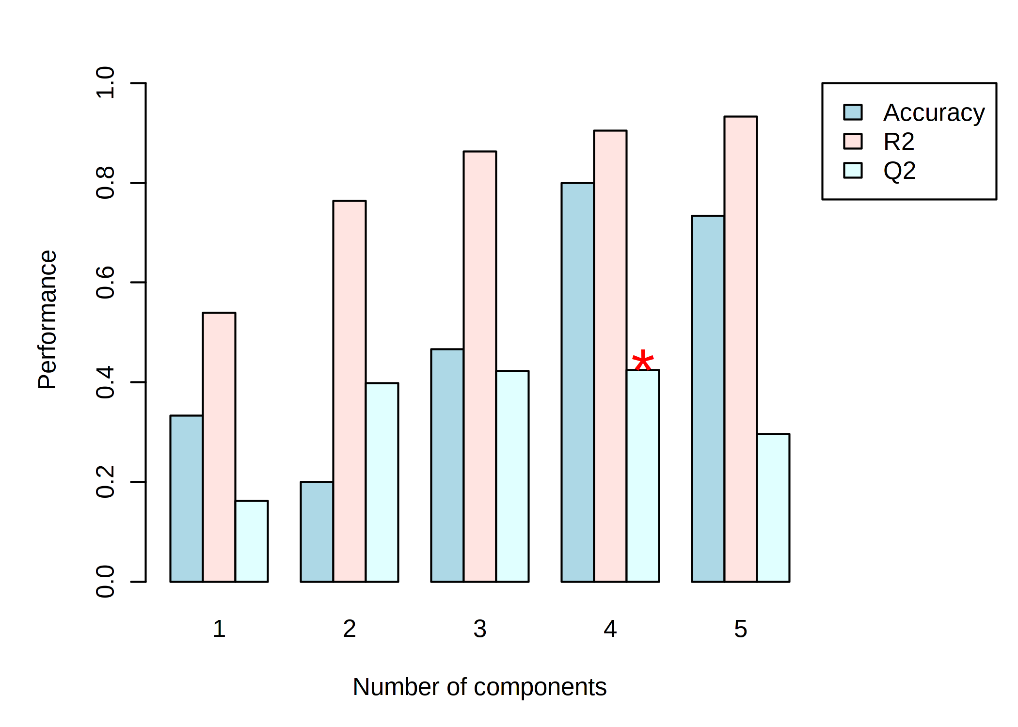
*

*Fig. S6. Orthogonal PLS-DA – Partial Lease Squares - Discriminant Analysis (PLS-DA) Amphipods exposed and unexposed to copper in Toilet Paper (including Initial Baseline amphipods) separation across substrates model overview.*

*
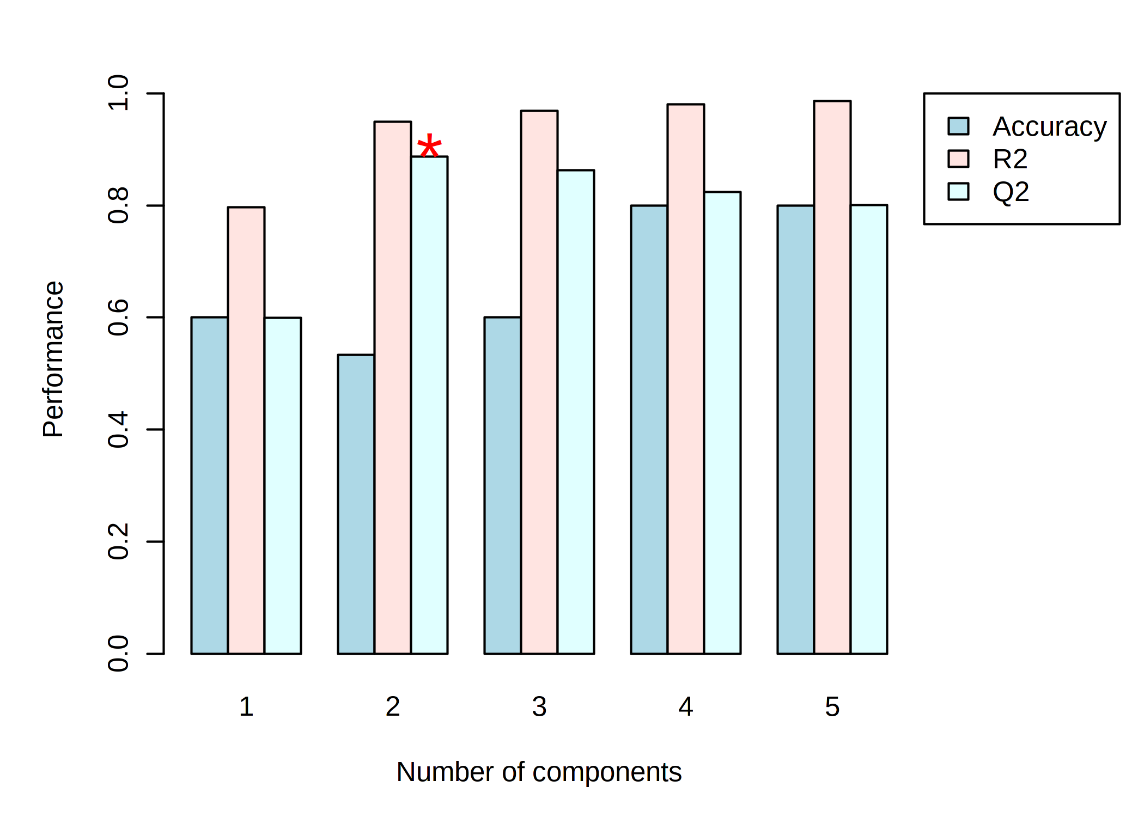
*

*Fig. S7. Partial Lease Squares - Discriminant Analysis (PLS-DA) Amphipods exposed and unexposed to copper in Gauze (including Initial Baseline amphipods) separation across substrates model overview.*

Pathway analysis reports listed below – reports collected from Metabolomic Data Analysis with MetaboAnalyst 5.0

# Metabolomic Data Analysis with MetaboAnalyst 5.0

**Name: Adult Amphipod Pathway analysis**

February 10, 2022

## Background

The Pathway Analysis module combines results from powerful pathway enrichment analysis with pathway topology analysis to help researchers identify the most relevant pathways involved in the conditions under study.

There are many commercial pathway analysis software tools such as Pathway Studio, MetaCore, or Ingenuity Pathway Analysis (IPA), etc. Compared to these commercial tools, the pathway analy- sis module was specifically developed for metabolomics studies. It uses high-quality KEGG metabolic pathways as the backend knowledgebase. This module integrates many well-established (i.e. univariate analysis, over-representation analysis) methods, as well as novel algorithms and concepts (i.e. Global Test, GlobalAncova, network topology analysis) into pathway analysis. Another feature is a Google-Map style interactive visualization system to deliver the analysis results in an intuitive manner.

## Data Input

The Pathway Analysis module accepts either a list of compound labels (common names, HMDB IDs or KEGG IDs) with one compound per row, or a compound concentration table with samples in rows and compounds in columns. The second column must be phenotype labels (binary, multi-group, or continuous). The table is uploaded as comma separated values (.csv).

## Compound Name Matching

The first step is to standardize the compound labels used in user uploaded data. This is a necessary step since these compounds will be subsequently compared with compounds contained in the pathway library. There are three outcomes from the step - exact match, approximate match (for common names only), and no match. Users should click the textbfView button from the approximate matched results to manually select the correct one. Compounds without match will be excluded from the subsequently pathway analysis.

**Table S7** shows the conversion results. Note: *1* indicates exact match, *2* indicates approximate match, and *0* indicates no match. A text file contain the result can be found the downloaded file *name map.csv*

Table S7: Result from Compound Name Mapping

| Query | Match | HMDB | PubChem | KEGG | SMILES |
| --- | --- | --- | --- | --- | --- |
| 1 Tetradecanoic acid | Myristic acid | HMDB0000806 | 11005 | C06424 | CCCCCCCCCCCCCC(=O)O |
| 2 Gluconic acid | Gluconic acid | HMDB0000625 | 10690 | C00257 | C([C@H]([C@H]([C@@H]([C@H](C(=O)O)O) |
| 3 Glucose | D-Glucose | HMDB0000122 | 5793 | C00221 | C([C@@H]1[C@H]([C@@H]([C@H](C(O1)O)O |
| 4 Succinic acid | Succinic acid | HMDB0000254 | 1110 | C00042 | C(CC(=O)O)C(=O)O |
| 5 Glutaric acid | Glutaric acid | HMDB0000661 | 743 | C00489 | C(CC(=O)O)CC(=O)O |
| 6 Octadecanoic acid | Stearic acid | HMDB0000827 | 5281 | C01530 | CCCCCCCCCCCCCCCCCC(=O)O |
| 7 Glyceryl monostearate | Glycerol 1-octadecanoate | HMDB0031075 | 24699 |  | CCCCCCCCCCCCCCCCCC(=O)OCC(CO)O |
| 8 Glycine | Glycine | HMDB0000123 | 750 | C00037 | C(C(=O)O)N |
| 9 Trehalose | Trehalose | HMDB0000975 | 7427 | C01083 | C([C@@H]1[C@H]([C@@H]([C@H]([C@H](O1 |

| 10 Palmitic acid | Palmitic acid | HMDB0000220 | 985 | C00249 | CCCCCCCCCCCCCCCC(=O)O |
| --- | --- | --- | --- | --- | --- |
| 11 Unidentified 3 | NA | NA | NA | NA | NA |
| 12 Unidentified 1 | NA | NA | NA | NA | NA |
| 13 Androsterone | Androsterone | HMDB0000031 | 5879 | C00523 | C[C@]12CCC(C[C@@H]1CC[C@@H]3[C@@H |
| 14 Unidentified 2 | NA | NA | NA | NA | NA |
| 15 Propanoic acid | Propionic acid | HMDB0000237 | 1032 | C00163 | CCC(=O)O |
| 16 Valine | L-Valine | HMDB0000883 | 6287 | C00183 | CC(C)[C@@H](C(=O)O)N |
| 17 Turanose | Turanose | HMDB0011740 | 5460935 | C19636 | C([C@@H]1[C@H]([C@@H]([C@H]([C@H](O1 |
| 18 Valeric acid | Valeric acid | HMDB0000892 | 7991 | C00803 | CCCCC(=O)O |
| 19 Myo inositol | myo-Inositol | HMDB0000211 |  | C00137 | O[C@H]1[C@H](O)[C@@H](O)[C@H](O)[C@H |
| 20 Elaidic acid | Elaidic acid | HMDB0000573 | 637517 | C01712 | CCCCCCCC/C=C*\*CCCCCCCC(=O)O |

## Pathway Analysis

In this step, users are asked to select a pathway library, as well as specify the algorithms for pathway enrichment analysis and pathway topology analysis.

### Pathway Library

There are 15 pathway libraries currently supported, with a total of 1173 pathways :

- - - Homo sapiens (human) [80]
    - Mus musculus (mouse) [82]
    - Rattus norvegicus (rat) [81]
    - Bos taurus (cow) [81]
    - Danio rerio (zebrafish) [81]
    - Drosophila melanogaster (fruit fly) [79]
    - Caenorhabditis elegans (nematode) [78]
    - Saccharomyces cerevisiae (yeast) [65]
    - Oryza sativa japonica (Japanese rice) [83]
    - Arabidopsis thaliana (thale cress) [87]
    - Escherichia coli K-12 MG1655 [87]
    - Bacillus subtilis [80]
    - Pseudomonas putida KT2440 [89]
    - Staphylococcus aureus N315 (MRSA/VSSA)[73]
    - Thermotoga maritima [57]

Your selected pathway library code is **dme** (KEGG organisms abbreviation).

### Over Representation Analysis

Over-representation analysis tests if a particular group of compounds is represented more than expected by chance within the user uploaded compound list. In the context of pathway analysis, we are testing if compounds involved in a particular pathway are enriched compared to random hits. MetPA offers two of the most commonly used methods for over-representation analysis:

- - - Fishers’Exact test
    - Hypergeometric Test

*Please note, MetPA uses one-tailed Fisher’s exact test which will give essentially the same result as the result calculated by the hypergeometric test.*

The selected over-representation analysis method is **Hypergeometric test**.

### Pathway Topology Analysis

The structure of biological pathways represent our knowledge about the complex relationships among molecules within a cell or a living organism. However, most pathway analysis algorithms fail to take structural information into consideration when estimating which pathways are significantly changed under conditions of study. It is well-known that changes in more important positions of a network will trigger a more severe impact on the pathway than changes occurred in marginal or relatively isolated positions.

The pathway topology analysis uses two well-established node centrality measures to estimate node importance - **degree centrality** and **betweenness centrality**. Degree centrality is defined as the number of links occurred upon a node. For a directed graph there are two types of degree: in-degree for links come from other nodes, and out-degree for links initiated from the current node. Metabolic networks are directed graph. Here we only consider the out-degree for node importance measure. It is assumed that nodes upstream will have regulatory roles for the downstream nodes, not vice versa. The betweenness centrality measures the number of shortest paths going through the node. Since the metabolic network is directed, we use the relative betweenness centrality for a metabolite as the importance measure. The degree centrality measure focuses more on local connectivities, while the betweenness centrality measure focuses more on global network topology. For more detailed discussions on various graph-based methods for analyzing biological networks, please refer to the article by Tero Aittokallio, T. et al. ^1^

*Please note, for comparison among different pathways, the node importance values calculated from centrality measures are further normalized by the sum of the importance of the pathway. Therefore, the total/maximum importance of each pathway is 1; the importance measure of each metabolite node is actually the percentage w.r.t the total pathway importance, and the pathway impact value is the cumulative percentage from the matched metabolite nodes.*

Your selected node importance measure for topological analysis is **relative betweenness centrality**.

## Pathway Analysis Result

The results from pathway analysis are presented graphically as well as in a detailed table.

A Google-map style interactive visualization system was implemented to facilitate data exploration. The graphical output contains three levels of view: **metabolome view**, **pathway view**, and **com- pound view**. Only the metabolome view is shown below. Pathway views and compound views are generated dynamically based on your interactions with the visualization system. They are available in your downloaded files.

^1^Tero Aittokallio and Benno Schwikowski. *Graph-based methods for analyzing networks in cell biology*, Briefings in Bioinformatics 2006 7(3):243-255


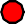

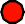

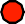

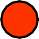

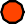

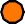


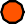

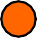


-log10(p)

0.8

1.0

1.2

1.4

####
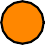

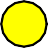

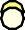
0.00 0.05 0.10 0.15 0.20 0.25 0.30


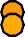

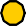

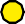


0.4

0.6

Pathway Impact

Figure S7: Summary of Pathway Analysis

The table below shows the detailed results from the pathway analysis. Since we are testing many pathways at the same time, the statistical p values from enrichment analysis are further adjusted for multiple testings. In particular, the **Total** is the total number of compounds in the pathway; the **Hits** is the actually matched number from the user uploaded data; the **Raw p** is the original p value calculated from the enrichment analysis; the **Holm p** is the p value adjusted by Holm-Bonferroni method; the **FDR p** is the p value adjusted using False Discovery Rate; the **Impact** is the pathway impact value calculated from pathway topology analysis.

Table S8: Result from Pathway Analysis

|  | Total | Expected | Hits | Raw p | -log10(p) | Holm adjust | FDR | Impact |
| --- | --- | --- | --- | --- | --- | --- | --- | --- |
| Propanoate metabolism | 21 | 0.32 | 2 | 3.77E-02 | 1.42E+00 | 1.00E+00 | 1.00E+00 | 0.00 |
| Ascorbate and aldarate metabolism | 6 | 0.09 | 1 | 8.71E-02 | 1.06E+00 | 1.00E+00 | 1.00E+00 | 0.00 |
| Valine, leucine and isoleucine biosynthe- | 8 | 0.12 | 1 | 1.15E-01 | 9.41E-01 | 1.00E+00 | 1.00E+00 | 0.00 |
| sis |  |  |  |  |  |  |  |  |
| Fatty acid biosynthesis | 43 | 0.65 | 2 | 1.34E-01 | 8.73E-01 | 1.00E+00 | 1.00E+00 | 0.02 |
| Aminoacyl-tRNA biosynthesis | 48 | 0.72 | 2 | 1.60E-01 | 7.95E-01 | 1.00E+00 | 1.00E+00 | 0.00 |
| Butanoate metabolism | 14 | 0.21 | 1 | 1.92E-01 | 7.16E-01 | 1.00E+00 | 1.00E+00 | 0.00 |
| Starch and sucrose metabolism | 14 | 0.21 | 1 | 1.92E-01 | 7.16E-01 | 1.00E+00 | 1.00E+00 | 0.01 |
| Pantothenate and CoA biosynthesis | 18 | 0.27 | 1 | 2.40E-01 | 6.19E-01 | 1.00E+00 | 1.00E+00 | 0.00 |
| Citrate cycle (TCA cycle) | 20 | 0.30 | 1 | 2.63E-01 | 5.79E-01 | 1.00E+00 | 1.00E+00 | 0.03 |
| Alanine, aspartate and glutamate | 23 | 0.35 | 1 | 2.97E-01 | 5.27E-01 | 1.00E+00 | 1.00E+00 | 0.00 |
| metabolism |  |  |  |  |  |  |  |  |
| Porphyrin and chlorophyll metabolism | 24 | 0.36 | 1 | 3.08E-01 | 5.12E-01 | 1.00E+00 | 1.00E+00 | 0.00 |
| Glyoxylate and dicarboxylate | 24 | 0.36 | 1 | 3.08E-01 | 5.12E-01 | 1.00E+00 | 1.00E+00 | 0.12 |
| metabolism |  |  |  |  |  |  |  |  |
| Glycolysis / Gluconeogenesis | 26 | 0.39 | 1 | 3.29E-01 | 4.83E-01 | 1.00E+00 | 1.00E+00 | 0.00 |
| Glutathione metabolism | 26 | 0.39 | 1 | 3.29E-01 | 4.83E-01 | 1.00E+00 | 1.00E+00 | 0.09 |
| Galactose metabolism | 27 | 0.41 | 1 | 3.39E-01 | 4.70E-01 | 1.00E+00 | 1.00E+00 | 0.00 |
| Phosphatidylinositol signaling system | 28 | 0.42 | 1 | 3.49E-01 | 4.57E-01 | 1.00E+00 | 1.00E+00 | 0.04 |
| Inositol phosphate metabolism | 28 | 0.42 | 1 | 3.49E-01 | 4.57E-01 | 1.00E+00 | 1.00E+00 | 0.12 |
| Glycine, serine and threonine metabolism | 30 | 0.45 | 1 | 3.69E-01 | 4.33E-01 | 1.00E+00 | 1.00E+00 | 0.29 |
| Fatty acid elongation | 37 | 0.56 | 1 | 4.35E-01 | 3.62E-01 | 1.00E+00 | 1.00E+00 | 0.00 |
| Fatty acid degradation | 38 | 0.57 | 1 | 4.44E-01 | 3.53E-01 | 1.00E+00 | 1.00E+00 | 0.00 |
| Valine, leucine and isoleucine degrada- | 38 | 0.57 | 1 | 4.44E-01 | 3.53E-01 | 1.00E+00 | 1.00E+00 | 0.00 |
| tion |  |  |  |  |  |  |  |  |

# Metabolomic Data Analysis with MetaboAnalyst 5.0

**Name: Juvenile Amphipod Pathway analysis**

February 10, 2022

## Background

The Pathway Analysis module combines results from powerful pathway enrichment analysis with pathway topology analysis to help researchers identify the most relevant pathways involved in the conditions under study.

There are many commercial pathway analysis software tools such as Pathway Studio, MetaCore, or Ingenuity Pathway Analysis (IPA), etc. Compared to these commercial tools, the pathway analy- sis module was specifically developed for metabolomics studies. It uses high-quality KEGG metabolic pathways as the backend knowledgebase. This module integrates many well-established (i.e. univariate analysis, over-representation analysis) methods, as well as novel algorithms and concepts (i.e. Global Test, GlobalAncova, network topology analysis) into pathway analysis. Another feature is a Google-Map style interactive visualization system to deliver the analysis results in an intuitive manner.

## Data Input

The Pathway Analysis module accepts either a list of compound labels (common names, HMDB IDs or KEGG IDs) with one compound per row, or a compound concentration table with samples in rows and compounds in columns. The second column must be phenotype labels (binary, multi-group, or continuous). The table is uploaded as comma separated values (.csv).

## Compound Name Matching

The first step is to standardize the compound labels used in user uploaded data. This is a necessary step since these compounds will be subsequently compared with compounds contained in the pathway library. There are three outcomes from the step - exact match, approximate match (for common names only), and no match. Users should click the textbfView button from the approximate matched results to manually select the correct one. Compounds without match will be excluded from the subsequently pathway analysis.

**Table S9** shows the conversion results. Note: *1* indicates exact match, *2* indicates approximate match, and *0* indicates no match. A text file contain the result can be found the downloaded file *name map.csv*

Table S9: Result from Compound Name Mapping

|  | Query | Match | HMDB | PubChem | KEGG | SMILES |
| --- | --- | --- | --- | --- | --- | --- |
| 1 | Octadecanoic acid | Stearic acid | HMDB0000827 | 5281 | C01530 | CCCCCCCCCCCCCCCCCC(=O)O |
| 2 | Glycerol | Glycerol | HMDB0000131 | 753 | C00116 | C(C(CO)O)O |
| 3 | Palmitic acid | Palmitic acid | HMDB0000220 | 985 | C00249 | CCCCCCCCCCCCCCCC(=O)O |
| 4 | Proline | L-Proline | HMDB0000162 | 145742 | C00148 | C1C[C@H](NC1)C(=O)O |
| 5 | Alanine | L-Alanine | HMDB0000161 | 5950 | C00041 | C[C@@H](C(=O)O)N |
| 6 | Unidentified 3 | NA | NA | NA | NA | NA |
| 7 | Unidentified 7 | NA | NA | NA | NA | NA |
| 8 | Sucrose | Sucrose | HMDB0000258 | 5988 | C00089 | C([C@@H]1[C@H]([C@@H]([C@H]([C@H](O1)O[C@]2([C@ |
| 9 | Unidentified 1 | NA | NA | NA | NA | NA |

| 10 | Glutamic acid | L-Glutamic acid | HMDB0000148 | 33032 | C00025 | C(CC(=O)O)[C@@H](C(=O)O)N |
| --- | --- | --- | --- | --- | --- | --- |
| 11 | Glutamine | L-Glutamine | HMDB0000641 | 5961 | C00064 | C(CC(=O)N)[C@@H](C(=O)O)N |
| 12 | Androst amine | NA | NA | NA | NA | NA |
| 13 | Glucose | D-Glucose | HMDB0000122 | 5793 | C00221 | C([C@@H]1[C@H]([C@@H]([C@H](C(O1)O)O)O)O)O |
| 14 | Talose | NA | NA | NA | NA | NA |
| 15 | Valine | L-Valine | HMDB0000883 | 6287 | C00183 | CC(C)[C@@H](C(=O)O)N |
|  |  |  |  |  |  |  |

## Pathway Analysis

In this step, users are asked to select a pathway library, as well as specify the algorithms for pathway enrichment analysis and pathway topology analysis.

### Pathway Library

There are 15 pathway libraries currently supported, with a total of 1173 pathways :

- Homo sapiens (human) [80]
  - - Mus musculus (mouse) [82]
    - Rattus norvegicus (rat) [81]
    - Bos taurus (cow) [81]
    - Danio rerio (zebrafish) [81]
    - Drosophila melanogaster (fruit fly) [79]
    - Caenorhabditis elegans (nematode) [78]
    - Saccharomyces cerevisiae (yeast) [65]
    - Oryza sativa japonica (Japanese rice) [83]
    - Arabidopsis thaliana (thale cress) [87]
    - Escherichia coli K-12 MG1655 [87]
    - Bacillus subtilis [80]
    - Pseudomonas putida KT2440 [89]
    - Staphylococcus aureus N315 (MRSA/VSSA)[73]
    - Thermotoga maritima [57]

Your selected pathway library code is **dme** (KEGG organisms abbreviation).

### Over Representation Analysis

Over-representation analysis tests if a particular group of compounds is represented more than expected by chance within the user uploaded compound list. In the context of pathway analysis, we are testing if compounds involved in a particular pathway are enriched compared to random hits. MetPA offers two of the most commonly used methods for over-representation analysis:

- - - Fishers’Exact test
    - Hypergeometric Test

*Please note, MetPA uses one-tailed Fisher’s exact test which will give essentially the same result as the result calculated by the hypergeometric test.*

The selected over-representation analysis method is **Hypergeometric test**.

### Pathway Topology Analysis

The structure of biological pathways represent our knowledge about the complex relationships among molecules within a cell or a living organism. However, most pathway analysis algorithms fail to take structural information into consideration when estimating which pathways are significantly changed under conditions of study. It is well-known that changes in more important positions of a network will trigger a more severe impact on the pathway than changes occurred in marginal or relatively isolated positions.

The pathway topology analysis uses two well-established node centrality measures to estimate node importance - **degree centrality** and **betweenness centrality**. Degree centrality is defined as the number of links occurred upon a node. For a directed graph there are two types of degree: in-degree for links come from other nodes, and out-degree for links initiated from the current node. Metabolic networks are directed graph. Here we only consider the out-degree for node importance measure. It is assumed that nodes upstream will have regulatory roles for the downstream nodes, not vice versa. The betweenness centrality measures the number of shortest paths going through the node. Since the metabolic network is directed, we use the relative betweenness centrality for a metabolite as the importance measure. The degree centrality measure focuses more on local connectivities, while the betweenness centrality measure focuses more on global network topology. For more detailed discussions on various graph-based methods for analyzing biological networks, please refer to the article by Tero Aittokallio, T. et al. ^1^

*Please note, for comparison among different pathways, the node importance values calculated from centrality measures are further normalized by the sum of the importance of the pathway. Therefore, the total/maximum importance of each pathway is 1; the importance measure of each metabolite node is actually the percentage w.r.t the total pathway importance, and the pathway impact value is the cumulative percentage from the matched metabolite nodes.*

Your selected node importance measure for topological analysis is **relative betweenness centrality**.

## Pathway Analysis Result

The results from pathway analysis are presented graphically as well as in a detailed table.

A Google-map style interactive visualization system was implemented to facilitate data exploration. The graphical output contains three levels of view: **metabolome view**, **pathway view**, and **com- pound view**. Only the metabolome view is shown below. Pathway views and compound views are generated dynamically based on your interactions with the visualization system. They are available in your downloaded files.

^1^Tero Aittokallio and Benno Schwikowski. *Graph-based methods for analyzing networks in cell biology*, Briefings in Bioinformatics 2006 7(3):243-255


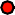

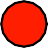

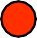

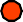

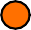

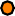

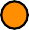

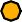


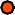

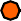

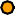

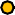

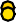


-log10(p)

1

2

3

4


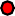


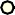


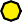

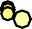


#### 0.0 0.2 0.4 0.6 0.8 1.0

Pathway Impact

Figure S8: Summary of Pathway Analysis

The table below shows the detailed results from the pathway analysis. Since we are testing many pathways at the same time, the statistical p values from enrichment analysis are further adjusted for multiple testings. In particular, the **Total** is the total number of compounds in the pathway; the **Hits** is the actually matched number from the user uploaded data; the **Raw p** is the original p value calculated from the enrichment analysis; the **Holm p** is the p value adjusted by Holm-Bonferroni method; the **FDR p** is the p value adjusted using False Discovery Rate; the **Impact** is the pathway impact value calculated from pathway topology analysis.

Table S10: Result from Pathway Analysis

|  | Total | Expected | Hits | Raw p | -log10(p) | Holm adjust | FDR | Impact |
| --- | --- | --- | --- | --- | --- | --- | --- | --- |
| Aminoacyl-tRNA biosynthesis | 48 | 0.45 | 5 | 3.23E-05 | 4.49E+00 | 2.61E-03 | 2.61E-03 | 0.00 |
| Nitrogen metabolism | 5 | 0.05 | 2 | 7.84E-04 | 3.11E+00 | 6.27E-02 | 1.95E-02 | 0.00 |
| D-Glutamine and D-glutamate | 5 | 0.05 | 2 | 7.84E-04 | 3.11E+00 | 6.27E-02 | 1.95E-02 | 1.00 |
| Metabolism |  |  |  |  |  |  |  |  |
| Alanine, aspartate and glutamate | 23 | 0.22 | 3 | 9.61E-04 | 3.02E+00 | 7.50E-02 | 1.95E-02 | 0.46 |
| metabolism |  |  |  |  |  |  |  |  |
| Arginine biosynthesis | 12 | 0.11 | 2 | 4.99E-03 | 2.30E+00 | 3.85E-01 | 8.09E-02 | 0.06 |
| Glyoxylate and dicarboxylate | 24 | 0.23 | 2 | 1.97E-02 | 1.71E+00 | 1.00E+00 | 2.65E-01 | 0.00 |
| metabolism |  |  |  |  |  |  |  |  |
| Galactose metabolism | 27 | 0.25 | 2 | 2.46E-02 | 1.61E+00 | 1.00E+00 | 2.85E-01 | 0.03 |
| Arginine and proline metabolism | 31 | 0.29 | 2 | 3.20E-02 | 1.50E+00 | 1.00E+00 | 3.24E-01 | 0.22 |
| Valine, leucine and isoleucine biosynthesis | 8 | 0.08 | 1 | 7.30E-02 | 1.14E+00 | 1.00E+00 | 6.57E-01 | 0.00 |
| Glycerolipid metabolism | 13 | 0.12 | 1 | 1.16E-01 | 9.35E-01 | 1.00E+00 | 8.41E-01 | 0.19 |
| Butanoate metabolism | 14 | 0.13 | 1 | 1.25E-01 | 9.05E-01 | 1.00E+00 | 8.41E-01 | 0.00 |
| Starch and sucrose metabolism | 14 | 0.13 | 1 | 1.25E-01 | 9.05E-01 | 1.00E+00 | 8.41E-01 | 0.04 |
| Pantothenate and CoA biosynthesis | 18 | 0.17 | 1 | 1.57E-01 | 8.03E-01 | 1.00E+00 | 9.81E-01 | 0.00 |
| Porphyrin and chlorophyll metabolism | 24 | 0.23 | 1 | 2.05E-01 | 6.89E-01 | 1.00E+00 | 1.00E+00 | 0.00 |
| Glycolysis / Gluconeogenesis | 26 | 0.24 | 1 | 2.20E-01 | 6.58E-01 | 1.00E+00 | 1.00E+00 | 0.00 |
| Glutathione metabolism | 26 | 0.24 | 1 | 2.20E-01 | 6.58E-01 | 1.00E+00 | 1.00E+00 | 0.04 |
| Fatty acid elongation | 37 | 0.35 | 1 | 2.99E-01 | 5.24E-01 | 1.00E+00 | 1.00E+00 | 0.00 |
| Fatty acid degradation | 38 | 0.36 | 1 | 3.06E-01 | 5.14E-01 | 1.00E+00 | 1.00E+00 | 0.00 |
| Valine, leucine and isoleucine degrada- | 38 | 0.36 | 1 | 3.06E-01 | 5.14E-01 | 1.00E+00 | 1.00E+00 | 0.00 |
| tion |  |  |  |  |  |  |  |  |
| Pyrimidine metabolism | 40 | 0.38 | 1 | 3.19E-01 | 4.96E-01 | 1.00E+00 | 1.00E+00 | 0.00 |
| Fatty acid biosynthesis | 43 | 0.40 | 1 | 3.39E-01 | 4.70E-01 | 1.00E+00 | 1.00E+00 | 0.02 |
| Purine metabolism | 63 | 0.59 | 1 | 4.58E-01 | 3.39E-01 | 1.00E+00 | 1.00E+00 | 0.00 |
|  |  |  |  |  |  |  |  |  |

# Metabolomic Data Analysis with MetaboAnalyst 5.0

**Name: Cellulose Substrate Exposure Pathway analysis**

February 10, 2022

## Background

The Pathway Analysis module combines results from powerful pathway enrichment analysis with pathway topology analysis to help researchers identify the most relevant pathways involved in the conditions under study.

There are many commercial pathway analysis software tools such as Pathway Studio, MetaCore, or Ingenuity Pathway Analysis (IPA), etc. Compared to these commercial tools, the pathway analy- sis module was specifically developed for metabolomics studies. It uses high-quality KEGG metabolic pathways as the backend knowledgebase. This module integrates many well-established (i.e. univariate analysis, over-representation analysis) methods, as well as novel algorithms and concepts (i.e. Global Test, GlobalAncova, network topology analysis) into pathway analysis. Another feature is a Google-Map style interactive visualization system to deliver the analysis results in an intuitive manner.

## Data Input

The Pathway Analysis module accepts either a list of compound labels (common names, HMDB IDs or KEGG IDs) with one compound per row, or a compound concentration table with samples in rows and compounds in columns. The second column must be phenotype labels (binary, multi-group, or continuous). The table is uploaded as comma separated values (.csv).

## Compound Name Matching

The first step is to standardize the compound labels used in user uploaded data. This is a necessary step since these compounds will be subsequently compared with compounds contained in the pathway library. There are three outcomes from the step - exact match, approximate match (for common names only), and no match. Users should click the textbfView button from the approximate matched results to manually select the correct one. Compounds without match will be excluded from the subsequently pathway analysis.

**Table S11** shows the conversion results. Note: *1* indicates exact match, *2* indicates approximate match, and *0* indicates no match. A text file contain the result can be found the downloaded file *name map.csv*

Table S11: Result from Compound Name Mapping

|  | Query | Match | HMDB | PubChem | KEGG | SMILES |
| --- | --- | --- | --- | --- | --- | --- |
| 1 | Glycerol | Glycerol | HMDB0000131 | 753 | C00116 | C(C(CO)O)O |
| 2 | Trehalose | Trehalose | HMDB0000975 | 7427 | C01083 | C([C@@H]1[C@H]([C@@H]([C@H]([C@H](O1)O[C@@H |
| 3 | Galactopyranoside | L-Galactose | HMDB0033704 | 6036 | C01825 | C([C@H]1[C@H]([C@H]([C@@H](C(O1)O)O)O)O)O |
| 4 | Lactic acid | L-Lactic acid | HMDB0000190 | 61503 | C00186 | C[C@@H](C(=O)O)O |
| 5  6 | Linolenic acid  Eicosenoic acid | Alpha-Linolenic acid  Eicosenoic acid | HMDB0001388  HMDB0002231 | 5280934  5282768 | C06427  C16526 | CC/C=C*\*C/C=C*\*C/C=C*\*CCCCCCCC(=O)O  CCCCCCCC/C=C*\*CCCCCCCCCC(=O)O |

## Pathway Analysis

In this step, users are asked to select a pathway library, as well as specify the algorithms for pathway enrichment analysis and pathway topology analysis.

### Pathway Library

There are 15 pathway libraries currently supported, with a total of 1173 pathways :

- - - Homo sapiens (human) [80]
    - Mus musculus (mouse) [82]
    - Rattus norvegicus (rat) [81]
    - Bos taurus (cow) [81]
    - Danio rerio (zebrafish) [81]
    - Drosophila melanogaster (fruit fly) [79]
    - Caenorhabditis elegans (nematode) [78]
    - Saccharomyces cerevisiae (yeast) [65]
    - Oryza sativa japonica (Japanese rice) [83]
    - Arabidopsis thaliana (thale cress) [87]
    - Escherichia coli K-12 MG1655 [87]
    - Bacillus subtilis [80]
    - Pseudomonas putida KT2440 [89]
    - Staphylococcus aureus N315 (MRSA/VSSA)[73]
    - Thermotoga maritima [57]

Your selected pathway library code is **dme** (KEGG organisms abbreviation).

### Over Representation Analysis

Over-representation analysis tests if a particular group of compounds is represented more than expected by chance within the user uploaded compound list. In the context of pathway analysis, we are testing if compounds involved in a particular pathway are enriched compared to random hits. MetPA offers two of the most commonly used methods for over-representation analysis:

- - - Fishers’Exact test
    - Hypergeometric Test

*Please note, MetPA uses one-tailed Fisher’s exact test which will give essentially the same result as the result calculated by the hypergeometric test.*

The selected over-representation analysis method is **Hypergeometric test**.

### Pathway Topology Analysis

The structure of biological pathways represent our knowledge about the complex relationships among molecules within a cell or a living organism. However, most pathway analysis algorithms fail to take structural information into consideration when estimating which pathways are significantly changed under conditions of study. It is well-known that changes in more important positions of a network will trigger a more severe impact on the pathway than changes occurred in marginal or relatively isolated positions.

The pathway topology analysis uses two well-established node centrality measures to estimate node importance - **degree centrality** and **betweenness centrality**. Degree centrality is defined as the number of links occurred upon a node. For a directed graph there are two types of degree: in-degree for links come from other nodes, and out-degree for links initiated from the current node. Metabolic networks are directed graph. Here we only consider the out-degree for node importance measure. It is assumed that nodes upstream will have regulatory roles for the downstream nodes, not vice versa. The betweenness centrality measures the number of shortest paths going through the node. Since the metabolic network is directed, we use the relative betweenness centrality for a metabolite as the importance measure. The degree centrality measure focuses more on local connectivities, while the betweenness centrality measure focuses more on global network topology. For more detailed discussions on various graph-based methods for analyzing biological networks, please refer to the article by Tero Aittokallio, T. et al. ^1^

*Please note, for comparison among different pathways, the node importance values calculated from centrality measures are further normalized by the sum of the importance of the pathway. Therefore, the total/maximum importance of each pathway is 1; the importance measure of each metabolite node is actually the percentage w.r.t the total pathway importance, and the pathway impact value is the cumulative percentage from the matched metabolite nodes.*

Your selected node importance measure for topological analysis is **relative betweenness centrality**.

## Pathway Analysis Result

The results from pathway analysis are presented graphically as well as in a detailed table.

A Google-map style interactive visualization system was implemented to facilitate data exploration. The graphical output contains three levels of view: **metabolome view**, **pathway view**, and **com- pound view**. Only the metabolome view is shown below. Pathway views and compound views are generated dynamically based on your interactions with the visualization system. They are available in your downloaded files.

^1^Tero Aittokallio and Benno Schwikowski. *Graph-based methods for analyzing networks in cell biology*, Briefings in Bioinformatics 2006 7(3):243-255


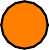

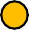


-log10(p)

0.9

1.0

1.1

1.2

1.3


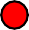


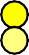


#### 0.00 0.05 0.10 0.15

Pathway Impact

Figure S9: Summary of Pathway Analysis

The table below shows the detailed results from the pathway analysis. Since we are testing many pathways at the same time, the statistical p values from enrichment analysis are further adjusted for multiple testings. In particular, the **Total** is the total number of compounds in the pathway; the **Hits** is the actually matched number from the user uploaded data; the **Raw p** is the original p value calculated from the enrichment analysis; the **Holm p** is the p value adjusted by Holm-Bonferroni method; the **FDR p** is the p value adjusted using False Discovery Rate; the **Impact** is the pathway impact value calculated from pathway topology analysis.

Table S12: Result from Pathway Analysis

|  | Total | Expected | Hits | Raw p | -log10(p) | Holm adjust | FDR | Impact |
| --- | --- | --- | --- | --- | --- | --- | --- | --- |
| alpha-Linolenic acid metabolism | 8 | 0.05 | 1 | 4.44E-02 | 1.35E+00 | 1.00E+00 | 1.00E+00 | 0.00 |
| Glycerolipid metabolism | 13 | 0.07 | 1 | 7.13E-02 | 1.15E+00 | 1.00E+00 | 1.00E+00 | 0.19 |
| Starch and sucrose metabolism | 14 | 0.08 | 1 | 7.66E-02 | 1.12E+00 | 1.00E+00 | 1.00E+00 | 0.01 |
| Pyruvate metabolism | 22 | 0.12 | 1 | 1.18E-01 | 9.28E-01 | 1.00E+00 | 1.00E+00 | 0.00 |
| Glycolysis / Gluconeogenesis | 26 | 0.15 | 1 | 1.38E-01 | 8.59E-01 | 1.00E+00 | 1.00E+00 | 0.00 |
| Galactose metabolism | 27 | 0.15 | 1 | 1.43E-01 | 8.44E-01 | 1.00E+00 | 1.00E+00 | 0.00 |

# Metabolomic Data Analysis with MetaboAnalyst 5.0

**Name: Gauze Substrate Exposure Pathway analysis**

February 10, 2022

## Background

The Pathway Analysis module combines results from powerful pathway enrichment analysis with pathway topology analysis to help researchers identify the most relevant pathways involved in the conditions under study.

There are many commercial pathway analysis software tools such as Pathway Studio, MetaCore, or Ingenuity Pathway Analysis (IPA), etc. Compared to these commercial tools, the pathway analy- sis module was specifically developed for metabolomics studies. It uses high-quality KEGG metabolic pathways as the backend knowledgebase. This module integrates many well-established (i.e. univariate analysis, over-representation analysis) methods, as well as novel algorithms and concepts (i.e. Global Test, GlobalAncova, network topology analysis) into pathway analysis. Another feature is a Google-Map style interactive visualization system to deliver the analysis results in an intuitive manner.

## Data Input

The Pathway Analysis module accepts either a list of compound labels (common names, HMDB IDs or KEGG IDs) with one compound per row, or a compound concentration table with samples in rows and compounds in columns. The second column must be phenotype labels (binary, multi-group, or continuous). The table is uploaded as comma separated values (.csv).

## Compound Name Matching

The first step is to standardize the compound labels used in user uploaded data. This is a necessary step since these compounds will be subsequently compared with compounds contained in the pathway library. There are three outcomes from the step - exact match, approximate match (for common names only), and no match. Users should click the textbfView button from the approximate matched results to manually select the correct one. Compounds without match will be excluded from the subsequently pathway analysis.

**Table S12** shows the conversion results. Note: *1* indicates exact match, *2* indicates approximate match, and *0* indicates no match. A text file contain the result can be found the downloaded file *name map.csv*

Table S12: Result from Compound Name Mapping

| Query | Match | HMDB | PubChem | KEGG | SMILES |
| --- | --- | --- | --- | --- | --- |
| 1 Glycerol | Glycerol | HMDB0000131 | 753 | C00116 | C(C(CO)O)O |
| 2 Glucose | D-Glucose | HMDB0000122 | 5793 | C00221 | C([C@@H]1[C@H]([C@@H]([C@H](C(O1)O)O)O)O)O |
| 3 Glycine | Glycine | HMDB0000123 | 750 | C00037 | C(C(=O)O)N |
| 4 Propanoic acid | Propionic acid | HMDB0000237 | 1032 | C00163 | CCC(=O)O |
| 5 Succinate acid | NA | NA | NA | NA | NA |
| 6 Palmitic acid | Palmitic acid | HMDB0000220 | 985 | C00249 | CCCCCCCCCCCCCCCC(=O)O |
| 7 Tetradecanoic acid | Myristic acid | HMDB0000806 | 11005 | C06424 | CCCCCCCCCCCCCC(=O)O |
| 8 Galactopyranoside | L-Galactose | HMDB0033704 | 6036 | C01825 | C([C@H]1[C@H]([C@H]([C@@H](C(O1)O)O)O)O)O |
| 9 Isopropanol | Isopropyl alcohol | HMDB0000863 | 3776 | C01845 | CC(C)O |

- 1. Linolenic acid Alpha-Linolenic acid HMDB0001388 5280934 C06427 CC/C=C C/C=C C/C=C CCCCCCCC(=O)O

*\ \ \*

- 1. Lactic acid L-Lactic acid HMDB0000190 61503 C00186 C[C@@H](C(=O)O)O

12 Trehalose Trehalose HMDB0000975 7427 C01083 C([C@@H]1[C@H]([C@@H]([C@H]([C@H](O1)O[C@@

13 Eicosenoic acid Eicosenoic acid HMDB0002231 5282768 C16526 CCCCCCCC/C=C*\*CCCCCCCCCC(=O)O

## Pathway Analysis

In this step, users are asked to select a pathway library, as well as specify the algorithms for pathway enrichment analysis and pathway topology analysis.

### Pathway Library

There are 15 pathway libraries currently supported, with a total of 1173 pathways :

- - - Homo sapiens (human) [80]
    - Mus musculus (mouse) [82]
    - Rattus norvegicus (rat) [81]
    - Bos taurus (cow) [81]
    - Danio rerio (zebrafish) [81]
    - Drosophila melanogaster (fruit fly) [79]
    - Caenorhabditis elegans (nematode) [78]
    - Saccharomyces cerevisiae (yeast) [65]
    - Oryza sativa japonica (Japanese rice) [83]
    - Arabidopsis thaliana (thale cress) [87]
    - Escherichia coli K-12 MG1655 [87]
    - Bacillus subtilis [80]
    - Pseudomonas putida KT2440 [89]
    - Staphylococcus aureus N315 (MRSA/VSSA)[73]
    - Thermotoga maritima [57]

Your selected pathway library code is **dme** (KEGG organisms abbreviation).

### Over Representation Analysis

Over-representation analysis tests if a particular group of compounds is represented more than expected by chance within the user uploaded compound list. In the context of pathway analysis, we are testing if compounds involved in a particular pathway are enriched compared to random hits. MetPA offers two of the most commonly used methods for over-representation analysis:

- - - Fishers’Exact test
    - Hypergeometric Test

*Please note, MetPA uses one-tailed Fisher’s exact test which will give essentially the same result as the result calculated by the hypergeometric test.*

The selected over-representation analysis method is **Hypergeometric test**.

### Pathway Topology Analysis

The structure of biological pathways represent our knowledge about the complex relationships among molecules within a cell or a living organism. However, most pathway analysis algorithms fail to take structural information into consideration when estimating which pathways are significantly changed under conditions of study. It is well-known that changes in more important positions of a network will trigger a more severe impact on the pathway than changes occurred in marginal or relatively isolated positions.

The pathway topology analysis uses two well-established node centrality measures to estimate node importance - **degree centrality** and **betweenness centrality**. Degree centrality is defined as the number of links occurred upon a node. For a directed graph there are two types of degree: in-degree for links come from other nodes, and out-degree for links initiated from the current node. Metabolic networks are directed graph. Here we only consider the out-degree for node importance measure. It is assumed that nodes upstream will have regulatory roles for the downstream nodes, not vice versa. The betweenness centrality measures the number of shortest paths going through the node. Since the metabolic network is directed, we use the relative betweenness centrality for a metabolite as the importance measure. The degree centrality measure focuses more on local connectivities, while the betweenness centrality measure focuses more on global network topology. For more detailed discussions on various graph-based methods for analyzing biological networks, please refer to the article by Tero Aittokallio, T. et al. ^1^

*Please note, for comparison among different pathways, the node importance values calculated from centrality measures are further normalized by the sum of the importance of the pathway. Therefore, the total/maximum importance of each pathway is 1; the importance measure of each metabolite node is actually the percentage w.r.t the total pathway importance, and the pathway impact value is the cumulative percentage from the matched metabolite nodes.*

Your selected node importance measure for topological analysis is **relative betweenness centrality**.

## Pathway Analysis Result

The results from pathway analysis are presented graphically as well as in a detailed table.

A Google-map style interactive visualization system was implemented to facilitate data exploration. The graphical output contains three levels of view: **metabolome view**, **pathway view**, and **com- pound view**. Only the metabolome view is shown below. Pathway views and compound views are generated dynamically based on your interactions with the visualization system. They are available in your downloaded files.

^1^Tero Aittokallio and Benno Schwikowski. *Graph-based methods for analyzing networks in cell biology*, Briefings in Bioinformatics 2006 7(3):243-255


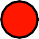

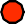

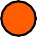

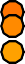

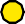

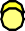

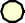


-log10(p)

0.4

0.6

0.8

1.0

1.2

1.4


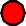


#### 0.00 0.05 0.10 0.15 0.20 0.25 0.30

Pathway Impact

Figure S10: Summary of Pathway Analysis

The table below shows the detailed results from the pathway analysis. Since we are testing many pathways at the same time, the statistical p values from enrichment analysis are further adjusted for multiple testings. In particular, the **Total** is the total number of compounds in the pathway; the **Hits** is the actually matched number from the user uploaded data; the **Raw p** is the original p value calculated from the enrichment analysis; the **Holm p** is the p value adjusted by Holm-Bonferroni method; the **FDR p** is the p value adjusted using False Discovery Rate; the **Impact** is the pathway impact value calculated from pathway topology analysis.

Table S13: Result from Pathway Analysis

|  | Total | Expected | Hits | Raw p | -log10(p) | Holm adjust | FDR | Impact |
| --- | --- | --- | --- | --- | --- | --- | --- | --- |
| Glycolysis / Gluconeogenesis | 26 | 0.29 | 2 | 3.26E-02 | 1.49E+00 | 1.00E+00 | 1.00E+00 | 0.00 |
| Fatty acid biosynthesis | 43 | 0.48 | 2 | 8.15E-02 | 1.09E+00 | 1.00E+00 | 1.00E+00 | 0.02 |
| alpha-Linolenic acid metabolism | 8 | 0.09 | 1 | 8.70E-02 | 1.06E+00 | 1.00E+00 | 1.00E+00 | 0.00 |
| Glycerolipid metabolism | 13 | 0.15 | 1 | 1.38E-01 | 8.61E-01 | 1.00E+00 | 1.00E+00 | 0.19 |
| Starch and sucrose metabolism | 14 | 0.16 | 1 | 1.48E-01 | 8.31E-01 | 1.00E+00 | 1.00E+00 | 0.01 |
| Propanoate metabolism | 21 | 0.24 | 1 | 2.14E-01 | 6.70E-01 | 1.00E+00 | 1.00E+00 | 0.00 |
| Pyruvate metabolism | 22 | 0.25 | 1 | 2.23E-01 | 6.52E-01 | 1.00E+00 | 1.00E+00 | 0.00 |
| Porphyrin and chlorophyll metabolism | 24 | 0.27 | 1 | 2.41E-01 | 6.19E-01 | 1.00E+00 | 1.00E+00 | 0.00 |
| Glyoxylate and dicarboxylate | 24 | 0.27 | 1 | 2.41E-01 | 6.19E-01 | 1.00E+00 | 1.00E+00 | 0.12 |
| metabolism |  |  |  |  |  |  |  |  |
| Glutathione metabolism | 26 | 0.29 | 1 | 2.58E-01 | 5.88E-01 | 1.00E+00 | 1.00E+00 | 0.09 |
| Galactose metabolism | 27 | 0.30 | 1 | 2.67E-01 | 5.74E-01 | 1.00E+00 | 1.00E+00 | 0.00 |
| Glycine, serine and threonine metabolism | 30 | 0.34 | 1 | 2.92E-01 | 5.35E-01 | 1.00E+00 | 1.00E+00 | 0.29 |
| Fatty acid elongation | 37 | 0.42 | 1 | 3.48E-01 | 4.59E-01 | 1.00E+00 | 1.00E+00 | 0.00 |
| Fatty acid degradation | 38 | 0.43 | 1 | 3.55E-01 | 4.50E-01 | 1.00E+00 | 1.00E+00 | 0.00 |
| Aminoacyl-tRNA biosynthesis | 48 | 0.54 | 1 | 4.27E-01 | 3.70E-01 | 1.00E+00 | 1.00E+00 | 0.00 |

# Metabolomic Data Analysis with MetaboAnalyst 5.0

**Name: Toilet paper Substrate Exposure Pathway analysis**

February 10, 2022

## Background

The Pathway Analysis module combines results from powerful pathway enrichment analysis with pathway topology analysis to help researchers identify the most relevant pathways involved in the conditions under study.

There are many commercial pathway analysis software tools such as Pathway Studio, MetaCore, or Ingenuity Pathway Analysis (IPA), etc. Compared to these commercial tools, the pathway analy- sis module was specifically developed for metabolomics studies. It uses high-quality KEGG metabolic pathways as the backend knowledgebase. This module integrates many well-established (i.e. univariate analysis, over-representation analysis) methods, as well as novel algorithms and concepts (i.e. Global Test, GlobalAncova, network topology analysis) into pathway analysis. Another feature is a Google-Map style interactive visualization system to deliver the analysis results in an intuitive manner.

## Data Input

The Pathway Analysis module accepts either a list of compound labels (common names, HMDB IDs or KEGG IDs) with one compound per row, or a compound concentration table with samples in rows and compounds in columns. The second column must be phenotype labels (binary, multi-group, or continuous). The table is uploaded as comma separated values (.csv).

## Compound Name Matching

The first step is to standardize the compound labels used in user uploaded data. This is a necessary step since these compounds will be subsequently compared with compounds contained in the pathway library. There are three outcomes from the step - exact match, approximate match (for common names only), and no match. Users should click the textbfView button from the approximate matched results to manually select the correct one. Compounds without match will be excluded from the subsequently pathway analysis.

**Table S14** shows the conversion results. Note: *1* indicates exact match, *2* indicates approximate match, and *0* indicates no match. A text file contains the result can be found the downloaded file *name map.csv*

Table S14: Result from Compound Name Mapping

Query Match HMDB PubChem KEGG SMILES Comment

1. Glycerol Glycerol HMDB0000131 753 C00116 C(C(CO)O)O 1
2. Lactic acid L-Lactic acid HMDB0000190 61503 C00186 C[C@@H](C(=O)O)O 1
3. Palmitic acid Palmitic acid HMDB0000220 985 C00249 CCCCCCCCCCCCCCCC(=O)O 1
4. Glycine Glycine HMDB0000123 750 C00037 C(C(=O)O)N 1
5. Eicosenoic acid Eicosenoic acid HMDB0002231 5282768 C16526 CCCCCCCC/C=C*\*CCCCCCCCCC(=O)O 1

## Pathway Analysis

In this step, users are asked to select a pathway library, as well as specify the algorithms for pathway enrichment analysis and pathway topology analysis.

### Pathway Library

There are 15 pathway libraries currently supported, with a total of 1173 pathways :

- - - Homo sapiens (human) [80]
    - Mus musculus (mouse) [82]
    - Rattus norvegicus (rat) [81]
    - Bos taurus (cow) [81]
    - Danio rerio (zebrafish) [81]
    - Drosophila melanogaster (fruit fly) [79]
    - Caenorhabditis elegans (nematode) [78]
    - Saccharomyces cerevisiae (yeast) [65]
    - Oryza sativa japonica (Japanese rice) [83]
    - Arabidopsis thaliana (thale cress) [87]
    - Escherichia coli K-12 MG1655 [87]
    - Bacillus subtilis [80]
    - Pseudomonas putida KT2440 [89]
    - Staphylococcus aureus N315 (MRSA/VSSA)[73]
    - Thermotoga maritima [57]

Your selected pathway library code is **dme** (KEGG organisms abbreviation).

### Over Representation Analysis

Over-representation analysis tests if a particular group of compounds is represented more than expected by chance within the user uploaded compound list. In the context of pathway analysis, we are testing if compounds involved in a particular pathway are enriched compared to random hits. MetPA offers two of the most commonly used methods for over-representation analysis:

- - - Fishers’Exact test
    - Hypergeometric Test

*Please note, MetPA uses one-tailed Fisher’s exact test which will give essentially the same result as the result calculated by the hypergeometric test.*

The selected over-representation analysis method is **Hypergeometric test**.

### Pathway Topology Analysis

The structure of biological pathways represents our knowledge about the complex relationships among molecules within a cell or a living organism. However, most pathway analysis algorithms fail to take structural information into consideration when estimating which pathways are significantly changed under conditions of study. It is well-known that changes in more important positions of a network will trigger a more severe impact on the pathway than changes occurred in marginal or relatively isolated positions.

The pathway topology analysis uses two well-established node centrality measures to estimate node importance - **degree centrality** and **betweenness centrality**. Degree centrality is defined as the number of links occurred upon a node. For a directed graph there are two types of degree: in-degree for links come from other nodes, and out-degree for links initiated from the current node. Metabolic networks are directed graph. Here we only consider the out-degree for node importance measure. It is assumed that nodes upstream will have regulatory roles for the downstream nodes, not vice versa. The betweenness centrality measures the number of shortest paths going through the node. Since the metabolic network is directed, we use the relative betweenness centrality for a metabolite as the importance measure. The degree centrality measure focuses more on local connectivities, while the betweenness centrality measure focuses more on global network topology. For more detailed discussions on various graph-based methods for analyzing biological networks, please refer to the article by Tero Aittokallio, T. et al. ^1^

*Please note, for comparison among different pathways, the node importance values calculated from centrality measures are further normalized by the sum of the importance of the pathway. Therefore, the total/maximum importance of each pathway is 1; the importance measure of each metabolite node is actually the percentage w.r.t the total pathway importance, and the pathway impact value is the cumulative percentage from the matched metabolite nodes.*

Your selected node importance measure for topological analysis is **relative betweenness centrality**.

## Pathway Analysis Result

The results from pathway analysis are presented graphically as well as in a detailed table.

A Google-map style interactive visualization system was implemented to facilitate data exploration. The graphical output contains three levels of view: **metabolome view**, **pathway view**, and **com- pound view**. Only the metabolome view is shown below. Pathway views and compound views are generated dynamically based on your interactions with the visualization system. They are available in your downloaded files.

^1^Tero Aittokallio and Benno Schwikowski. *Graph-based methods for analyzing networks in cell biology*, Briefings in Bioinformatics 2006 7(3):243-255


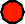

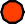


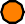

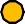


-log10(p)

0.9

1.0

1.1

1.2

####
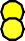

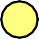

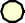
0.00 0.05 0.10 0.15 0.20 0.25 0.30

0.7

0.8

Pathway Impact

Figure S11: Summary of Pathway Analysis

The table below shows the detailed results from the pathway analysis. Since we are testing many pathways at the same time, the statistical p values from enrichment analysis are further adjusted for multiple testings. In particular, the **Total** is the total number of compounds in the pathway; the **Hits** is the actually matched number from the user uploaded data; the **Raw p** is the original p value calculated from the enrichment analysis; the **Holm p** is the p value adjusted by Holm-Bonferroni method; the **FDR p** is the p value adjusted using False Discovery Rate; the **Impact** is the pathway impact value calculated from pathway topology analysis.

Table 2: Result from Pathway Analysis

|  | Total | Expected | Hits | Raw p | -log10(p) | Holm adjust | FDR | Impact |
| --- | --- | --- | --- | --- | --- | --- | --- | --- |
| Glycerolipid metabolism | 13 | 0.06 | 1 | 5.97E-02 | 1.22E+00 | 1.00E+00 | 1.00E+00 | 0.19 |
| Pyruvate metabolism | 22 | 0.10 | 1 | 9.94E-02 | 1.00E+00 | 1.00E+00 | 1.00E+00 | 0.00 |
| Porphyrin and chlorophyll metabolism | 24 | 0.11 | 1 | 1.08E-01 | 9.67E-01 | 1.00E+00 | 1.00E+00 | 0.00 |
| Glyoxylate and dicarboxylate | 24 | 0.11 | 1 | 1.08E-01 | 9.67E-01 | 1.00E+00 | 1.00E+00 | 0.12 |
| metabolism |  |  |  |  |  |  |  |  |
| Glycolysis / Gluconeogenesis | 26 | 0.12 | 1 | 1.17E-01 | 9.33E-01 | 1.00E+00 | 1.00E+00 | 0.00 |
| Glutathione metabolism | 26 | 0.12 | 1 | 1.17E-01 | 9.33E-01 | 1.00E+00 | 1.00E+00 | 0.09 |
| Galactose metabolism | 27 | 0.13 | 1 | 1.21E-01 | 9.18E-01 | 1.00E+00 | 1.00E+00 | 0.00 |
| Glycine, serine and threonine metabolism | 30 | 0.14 | 1 | 1.33E-01 | 8.75E-01 | 1.00E+00 | 1.00E+00 | 0.29 |
| Fatty acid elongation | 37 | 0.17 | 1 | 1.62E-01 | 7.89E-01 | 1.00E+00 | 1.00E+00 | 0.00 |
| Fatty acid degradation | 38 | 0.18 | 1 | 1.67E-01 | 7.78E-01 | 1.00E+00 | 1.00E+00 | 0.00 |
| Fatty acid biosynthesis | 43 | 0.20 | 1 | 1.87E-01 | 7.29E-01 | 1.00E+00 | 1.00E+00 | 0.02 |
| Aminoacyl-tRNA biosynthesis | 48 | 0.23 | 1 | 2.06E-01 | 6.85E-01 | 1.00E+00 | 1.00E+00 | 0.00 |
